# Astrocytic 5-HT_1A_ receptor mediates age-dependent hippocampal LTD and fear memory extinction in male mice

**DOI:** 10.1038/s12276-024-01285-0

**Published:** 2024-08-01

**Authors:** Qian-Yun Wu, Lian-Hong Lin, Kun Lu, Si-Fu Deng, Wei-Min Li, Yuan Xu, Bin Zhang, Ji-Hong Liu

**Affiliations:** 1 Department of Psychiatry, Institute of Brain Disease, Nanfang Hospital, Southern Medical University, Guangzhou, 510515 China; 2 Guangdong-Hong Kong-Macao Greater Bay Area Center for Brain Science and Brain-Inspired Intelligence, Guangzhou, 510515 China; 3 Department of Pediatric Orthopaedic, Zhengzhou Orthopaedics Hospital, Zhengzhou, 450052 China

**Keywords:** Long-term depression, Extinction

## Abstract

NMDA receptor-dependent long-term depression (LTD) in the hippocampus is a well-known form of synaptic plasticity that has been linked to different cognitive functions. Although the underlying mechanisms remain unclear, this form of LTD cannot be induced by low-frequency stimulation (LFS) in adult mice. In this study, we found that LFS-induced LTD was not easily induced in adult animals and was age dependent. Interestingly, the level of the 5-HT_1A_ receptor was correspondingly increased and exhibited an inverse correlation with the magnitude of LFS-LTD during development. Knockout or pharmacological inhibition of the 5-HT_1A_ receptor reversed impaired LFS-LTD in adult mice (P60), while activation or inhibition of this receptor disturbed or enhanced LFS-LTD in adolescent mice (P21), respectively. Furthermore, the astrocytic 5-HT_1A_ receptor in the hippocampus predominantly mediated age-dependent LFS-LTD through enhancing GABAergic neurotransmission. Finally, fear memory extinction differed among the above conditions. These observations enrich our knowledge of LTD at the cellular level and suggest a therapeutic approach for LTD-related psychiatric disorders.

## Fear memory extinction varies with 5-HT1A receptor modulation

Understanding how our brains learn and remember is intriguing. As we age, our learning and memory abilities can alter, and scientists are trying to understand why. A recent study investigates this by studying a specific brain receptor, the 5-HT1A receptor, and its effect on learning and memory in mice. The team focused on a process called long-term depression. They found that the ability to induce LTD changes with age and that the 5-HT1A receptor plays a key role in this. They discovered that the activity of 5-HT1A receptors in certain brain cells, astrocytes, is necessary for LTD and influences fear memory extinction. The researchers conclude that the 5-HT1A receptor in astrocytes plays a crucial role in regulating learning and memory processes related to LTD. This discovery could lead to new treatments for memory-related disorders.

This summary was initially drafted using artificial intelligence, then revised and fact-checked by the author.

## Introduction

Long-term depression (LTD) in the CNS has been the subject of intense investigation as a mechanism that may be involved in learning and memory and in various pathological conditions^1–3^. NMDA receptor-dependent LTD in the hippocampus is a well-known form of synaptic plasticity that has been linked to different cognitive functions^4,5^. Hippocampal LTD can be experimentally induced by several different types of electrical and pharmacological stimulation methods. The most commonly used method for inducing LTD involves prolonged low-frequency stimulation (LFS) at 0.5–5 Hz^6–9^. Despite considerable progress in understanding the cellular and molecular mechanisms underlying LTD, LTD is difficult to elicit and less robust in hippocampal slices from adult animals than in slices from young animals^10–17^. However, the mechanisms underlying the age-related decrease in the magnitude of LTD remain elusive.

The serotonergic (5-HT) system is implicated in the neurobiological control of learning and memory and synaptic plasticity^18,19^. This system matures during early postnatal development, during which time it plays an important role in establishing circuits that mediate synaptic plasticity^20^. Once the 5-HT system has matured, it is well positioned for shape development^20^. Within the 5-HT system, signaling through the inhibitory serotonergic 1 A (5-HT_1A_) receptor is required for the normal development of circuits that subserve brain functions in mice^20–23^. The 5-HT_1A_ receptor is an inhibitory G protein-coupled receptor expressed both in serotonergic neurons and in target areas receiving serotonergic innervation^24,25^ and has been reported to play a crucial role in brain dysfunction, including alcoholism, cocaine abuse, Alzheimer’s disease, and schizophrenia^26–32^. These disorders have deleterious effects on activity-evoked synaptic plasticity, which is essential for neuronal survival and function. Furthermore, synaptic plasticity is of prime importance in early brain development, the impairment of which may be involved in the dysfunctions mentioned above, except for Alzheimer’s disease. Collectively, these findings strongly indicate that 5-HT_1A_ receptor-mediated molecular signaling may play an important role in the formation of crucial neuronal connections in the developing brain. However, despite the importance of the 5-HT_1A_ receptor in brain development, there is little information about the role of the 5-HT_1A_ receptor in LTD.

In this study, we investigated the relationship between the 5-HT_1A_ receptor and age-dependent LTD and found that the receptor functioned to modulate age-dependent LTD induction. Furthermore, we showed that the activity of 5-HT_1A_ receptors in astrocytes was required for hippocampal LFS-LTD and modulated fear memory extinction in vivo. In addition, the importance of the 5-HT_1A_ receptor on LTD induction emerged from maintaining the excitation/inhibition synaptic balance in the CA1 network of the hippocampus.

## Materials and methods

### Animals

In behavior, sex differences occur. The emergence of the estrous cycle in female mice is a contributing factor to the results of behavioral tests. Therefore, male C57BL/6J mice, aldh1-CreER^T2^ mice, CamkII-Cre mice, GAD-Cre mice and Sert-Cre mice aged P21 or 2 months (or any other age, if necessary) were used and housed in standard laboratory cages at 24 ± 1 °C. The number of mice used in each experiment is described in the corresponding figure legends.

According to our previous studies, by comparing the expression patterns of Cre recombinase between five widely used transgenic lines (hGfap-CreER^T2^, Glast-CreER^T2^, Cx30-CreER^T2^, Fgfr3-iCreER^T2^ and aldh1l1-CreER^T2^), we found that the aldh1l1-CreERT2 mouse line was the best model for studying astrocytes because it specifically targets astrocytes^33–35^. Aldh1l1-CreER^T2^ knock-in mice were generated via a CRISPR/Cas9 system using Cas9 mRNA, sgRNA (CCAGGTCTTGTCCCCAATACTGG) and a donor, which were coinjected into C57BL/6J zygotes by microinjection. Then, these zygotes were transplanted into pseudopregnant mice. Direct Cas9 endonuclease cleavage of the sgRNA occurs near the termination codon and creates a double-strand break (DSB). These breaks are subsequently repaired and result in a T2A-CreER^T2^ insertion before the stop codon of the Aldh1l1 gene. Tamoxifen was used to induce CreER^T2^-mediated recombination. Other mice (CamkII-Cre, GAD-Cre and Sert-Cre) were not of the Cre-ER type.

Before the experiments, mice were acclimatized to the new housing conditions for at least 1 week. The rats were housed five per cage under an artificial 12-h light/dark cycle (lights on from 8:00 A.M. to 8:00 P.M.) with ad libitum access to water and standard laboratory food at all times. All experimental procedures in this study were performed within the Chinese Council on Animal Care Guidelines for the Care and Use of Laboratory Animals.

### Reagents

Tamoxifen (Sigma‒Aldrich, #T5648), used to induce the expression of the Cre-ER^T2^ fusion protein, was dissolved in 10% ethanol/90% sunflower oil (Sigma‒Aldrich, #S5007) (v/v) at a final concentration of 10 mg/ml. Adult mice (P60) were intraperitoneally (i.p.) injected with tamoxifen (3 mg/40 g body weight) once a day for 7 consecutive days, according to our previous studies^33–36^. Experiments were performed two weeks after the last dose of tamoxifen. 8-OH-DPAT and WAY-100635 were purchased from Sigma (St. Louis, MO). (S)-3,5-Dihydroxyphenylglycine (DHPG) was purchased from Ascent Scientific (Bristol, UK). When necessary, the chemicals were dissolved in dimethyl sulfoxide (DMSO, Sigma), and the concentration of DMSO (Sigma) that was used for the solution was <0.1%.

### Slice preparation

Coronal hippocampal slices were prepared from male mice of different ages. First, each mouse was anesthetized with 1% pentobarbital sodium (Sigma, #P3761) before decapitation. The skull was opened, the brain quickly (within 1 min) removed and submerged in ice-cold ACSF composed of (in mM) 120 NaCl, 2.5 KCl, 1.2 NaH_2_PO_4_, 2.0 CaCl_2_, 2.0 MgSO_4_, 26 NaHCO_3_, and 10 glucose continuously bubbled with 95% O_2_ and 5%CO_2_, pH 7.4. After the brain was cooled in ACSF, it was trimmed and glued to the stage of a vibratome (Leica VT 1000 S, Germany) and immersed in a bath of ice-cold oxygenated ACSF. The hippocampus was dissected and cut into 300-µm-thick transverse slices. In most cases, slices containing the hippocampus in the transverse plane were selected. The hippocampal slices were transferred to a holding chamber with oxygenated ACSF at 34 °C for 30 min for recovery and then maintained at room temperature (25 ± 1 °C) for an additional 2–8 h before experimental recording. Slices were withdrawn from the holding chamber as needed and placed in a low-volume (2 ml) submerged recording chamber, where they were continuously perfused at a rate of 2.5 ml/min with standard ACSF. The recording chamber was kept at a temperature of 33 ± 1 °C by an automatic temperature controller (Warner TC-324B). After the slices were transferred from the holding chamber to the recording chamber, a minimum 30-min period was allowed for recovery.

### Electrophysiological recording

Field excitatory postsynaptic potentials (fEPSPs) were evoked in the stratum radiatum of CA1 by stimulation of Schaffer collaterals with two concentric bipolar stimulating electrodes (25 μm pole separation; FHC, ME) and recorded with ACSF-filled glass pipettes (1–5 MΩ) using Axoclamp-700B amplifiers and a Digidata 1440 analog-to-digital converter (Molecular Devices, Sunnyvale, CA). The test stimuli consisted of monophasic 100-µs pulses of constant current delivered by stimulus isolation units. fEPSPs were digitized (3 kHz), filtered at 10 kHz (eight-pole Bessel filter), analyzed on-line, and stored on computers using pClamp 10 software (Molecular Devices, Sunnyvale, CA).

The pulse intensity was adjusted to 25% of the maximum amplitude in all experiments. fEPSPs for which the 25% amplitude was ≥1 mV were used for data analysis. The strength of synaptic transmission was determined by measuring the initial (20–70% rising phase) slope of the fEPSP. All drugs were dissolved in ACSF and applied by switching the perfusion from control ACSF to drug-containing ACSF. During each recording, baseline synaptic transmission was monitored for 10 min before drug administration. The average fEPSP slopes during the 20 min prior to the induction of LTD or LTP were taken as the baseline, and all values were normalized to this baseline. The stimulus frequency at baseline was 0.033 Hz.

LTD was induced by LFS, which consisted of 900 pulses delivered at 1 Hz^5^. PP-LFS consisted of 900 paired pulses (40 ms intervals) at 1 Hz for 15 min. PP-LFS was repeated two times, 10 min apart. LTP was induced by 1 train of 100 Hz HFS stimulation lasting 1 s. PPF was studied by applying pairs of stimuli at varying interpulse intervals (20–200 ms). The slope of the response to the second pulse (P2) was averaged over 5–10 trials and divided by the average slope or amplitude of the response to the first pulse (P1) to obtain a ratio (P2/P1).

Whole-cell patch-clamp recording was performed on neurons in the hippocampal CA1 region. The pipettes were pulled by a micropipette puller (P-97, Sutter instrument) with a resistance of 3–7 MΩ. Recordings were made with a MultiClamp 700B amplifier and 1440 A digitizer (Molecular Devices). For sEPSC recording, pyramidal neurons were held at −70 mV in the presence of bicuculline methiodide (BMI, 20 μM) with a pipette solution containing (in mM) 130 K-gluconate, 20 KCl, 10 HEPES buffer, 4 Mg-ATP, 0.3 Na-GTP, 10 disodium phosphocreatine and 0.2 EGTA (pH 7.40, 285 mOsm). When recording sIPSCs, the holding potentials were −70 mV in the presence of kynurenic acid (1 mM), and pipettes were filled with an intracellular solution containing (in mM) 35 K-gluconate, 100 KCl, 2 EGTA, 10 HEPES, 5 NaCl, 0.1 NaGTP, 2 MgATP, and 5 QX-314 (pH 7.35, 285 mOsm).

### Western blot analysis

Western blotting was performed as described previously^35,37^. Briefly, the protein concentration was measured using a bicinchoninic acid (BCA) protein assay kit (#23227, Thermo, MA, USA). The samples were mixed with 2× sodium dodecyl sulfate (SDS) loading buffer, boiled for 10 min, and loaded onto 10% or 4–20% gradient polyacrylamide-SDS gels. Proteins were then transferred to a PVDF membrane (Millipore, Billerica, MA, USA) for 2 h at 350 mA, and the membranes were incubated in Odyssey Blocking Buffer (LICOR) for 2 h at room temperature. After overnight incubation with primary antibodies (polyclonal rabbit anti-5-HT_1A_R, 1:500) at 4 °C, the blots were washed three times in TBS containing 0.1% Tween-20 for 15 min and then incubated with peroxidase- or IRDye-conjugated secondary antibodies for 1 h in TBS supplemented with 0.1% Tween-20 at room temperature. Immunoreactivity was detected by chemiluminescence using an enhanced chemiluminescence (ECL) reagent and an LICOR imaging system.

### Immunofluorescence

Mice were anesthetized using 1% pentobarbital sodium (Sigma, #P3761) and transfused with saline, followed by 4% formaldehyde from the base of the left ventricle. The brains were cut into 40-μm-thick sections using a freezing microtome (Leica). The sections were washed with phosphate-buffered saline (PBS) and treated with 1% Triton-100, followed by incubation with goat serum and primary antibodies [mouse anti-GFAP (3670 S; 1:500, Cell Signaling Technology), mouse anti-NeuN (24307; 1:500, Cell Signaling Technology), and mouse anti-GAD67 (1:500; MAB5406; Millipore)] at 4 °C overnight. Sections were then incubated with the corresponding fluorescence-conjugated secondary antibodies [Alexa Fluor 488 (1:500; A11034; Invitrogen) or Alexa Fluor 594 (1:500; A11005; Invitrogen)] at room temperature for 1 h. The sections were then mounted using Fluoroshield mounting medium with 4’,6-diamidino-2-phenylindole (DAPI; ab104139; Abcam, Cambridge, MA, USA). Images with fluorescence were captured by fluorescence microscopy (Nikon).

### Cell counting

Five to eight sections for each brain area were obtained from each mouse, and three or five mice were used per experiment. The sections were blinded for analysis. The “Cell Counter” plugin in ImageJ 1.50i software was used for cell counting. The presence of DAPI labeling was required to identify each cell. The specificity of the virus that was injected into Cre mice was defined as the number of double-fluorescent cells/the total number of GFP^+^ cells. The specificity of Cre recombinase expression in the transgenic mouse line was defined as the number of double-fluorescent cells/the total number of GFP^+^ cells. The number of double-fluorescent cells/the total number of marker^+^ cells was used to define the mean efficiency of Cre recombinase expression.

### Microdialysis

The mice were deeply anesthetized and placed into a stereotaxic apparatus (Stoelting). A guide cannula (CMA/7, CMA/Microdialysis) was implanted into the hippocampus (AP = −2.0 mm; ML = 1.2 mm; DV = 1.2 mm). A microdialysis probe (CMA/7, membrane length: 1–2 mm, molecular weight cutoff: 6000 Da, outer diameter: 0.24 mm) was inserted through the guide cannula and connected to a syringe pump (CMA 402). The ASCF was continuously perfused through the microdialysis probe at a constant flow rate of 1 µl/min, and sampling was performed 1 h after the insertion of the probe. Two samples (30 µl each) were automatically collected from each mouse using the CMA 142 microfraction collector every 30 min for 60 min. To decrease the rate of background monoamine hydrolysis, each sample collection tube was pretreated with antioxidative agents (8 µl), including 100 mM acetic acid, 0.27 mM Na2EDTA and 12.5 μM ascorbic acid (pH 3.2).

### Quantitative real-time PCR

As previously reported^36,38^, brain samples were dissected and prepared after selection. Separate tissue samples were stored immediately in TRIzol (Invitrogen), and RNA was extracted according to the manufacturer’s directions. Genomic DNA was removed by gDNA eraser treatment (Takara), and 1.0 μg of RNA was used for first-strand complementary DNA (cDNA) synthesis (Takara). Quantitative reverse transcriptase PCR (qRT‒PCR) was performed on a Stratagene Mx3000P thermal cycler using Universal qRT‒PCR master mix for the indicated genes (Takara). The following primers were designed and synthesized:

*NRG1*, sense 5′-ACCAGCCATCTCATAAAGTGCG-3′, antisense 5′-TTGACGGGTTTGACAGGTCC-3′; *ErbB2*, sense 5′-TTGGTGGGCAGGTAGGTGAGTT-3′, antisense 5′-CCTGCCAGTCCCGAGACCCACCT-3′; *ErbB3*, sense 5′-GTCTGTGTGACCCACTGCAACT-3′, antisense 5′-GGGTGGCAGGAGAAGCATT-3′; *ErbB4*, sense 5′-AGTGGTCTGTCATTGCTTATCCTC-3′, antisense 5′-CTGTTGTCCGTGATGTAGATATTGC-3′; *BDNF*, sense 5′- AAAACCA TAAGGACGCGGACTT-3′, antisense 5′-GAGGCTCCAAAGG CACTTGA-3′; *GDNF*, sense 5′- TGACTCCAATATGCCTGAAG ATTATC-3′, antisense 5′-TCAGTCTTTTAATGGTGGCTTGAA- 3′; *IGF-1*, sense 5′- CCCGTCCCTATCGACAAACA-3′, antisense 5′-TTCCTGCACTTCCTCTACTTGTGT-3′; *VEGF*, sense 5′-GCAGGCTGCTACGATGA-3′, antisense 5′-TT GATCCGCATGATCTGCAT-3′; *FGF-2*, sense 5.

### Virus generation and stereotaxic injections

The recombinant AAV vectors were serotyped with AAV5 coat proteins and packaged by Shanghai Sunbio Medical Biotechnology (Shanghai, China). Viral titers were 2 × 10^12^ particles/ml. For in vivo viral injections, viral vectors were targeted to the hippocampal CA1 region (AP = −2.0 mm; ML = ± 1.6 mm; DV = −1.5 mm). Specifically, a Hamilton syringe fitted with a 33-gauge needle was filled with 1.5 µl of virus. The needle was lowered into the CA1 region, and 0.25 µl of virus was delivered over 2.5 min. The injection needle was withdrawn 5 min after infusion. Mice were used 3 weeks after AAV injection.

### Fluorescence-activated cell sorting (FACS)-droplet digital polymerase chain reaction (ddPCR)

As described previously^35^, brain slices containing the hippocampus were prepared using the standard methods for electrophysiology experiments as described above. The slices were blocked in a cocktail of D(−)-2-amino-5-phosphonovaleric acid (AP5), 6-cyano-7-nitroquinoxaline-2,3-dione (CNQX), and tetrodotoxin (TTX) to prevent excitotoxic cell death and then treated with the Papain dissociation system (Worthington) following the manufacturer’s instructions. Then, the cells were immediately loaded and sorted via FACS using the Beckman MoFlo XDP Cell Sorter system. The sorted ACSA^+^ or ACSA^−^ (ACSA-2: astrocyte cell surface antigen-2, specifically expressed on astrocytes) cells were enriched by centrifugation (1000 g, 3 min). After FACS, the total RNA from the sorted cells was extracted with an RNeasy Micro kit (QIAGEN, #74004), and the RNA quantity was assessed using a NanoDrop-1000. For droplet digital PCR, total RNA was reverse transcribed and amplified by a Discover-sc WTA Kit V2 (Vazyme, V2 N711-03) and PrimeScript^TM^ RT reagent kit (TaKaRa, #RR037A). Droplet digital PCR was performed with QX200 ddPCR EvaGreen Supermix (Bio-Rad, #186-4033), and 18S RNA served as an internal control. PCRs were detected using a QX200 Droplet Digital PCR System (Bio-Rad, CA). The analysis was performed using QuantaSoft software (Bio-Rad, v 1.7.4.0917). The 5-HT_1A_R and 18S mRNA levels were examined with the following primers: 5-HT_1A_R, forward: ACCCCAACGAGTGCACCATCAG, reverse: GCAGGCGGGGACATAGGAG; 18S, forward: A GTTCCAGCACATTTTGAG, reverse: TCATCCTCCGTGAGTT CTCCA.

### Behavioral analysis

The mice were handled by investigators for three days before any behavioral testing. The number of investigators handling the isolated animals during weekly cage changes was kept to a minimum.

The contextual fear conditioning test was conducted as reported previously to evaluate fear memory processes^35,37^. Mice were first habituated to the behavioral room and were then allowed to freely explore the apparatus (MED-VFC-NIR-M; Med Associates) for 3 min. During training, the mice were placed in conditioning chamber A and exposed to tone-foot-shock pairings (tone, 30 s, 80 dB; foot shock, 1 s, 0.75 mA), with an interval of 80 s. Mice were presented with four tone-shock pairings. After three days, the mice were returned to chamber A to evaluate contextual fear learning. Freezing during training and testing was scored using Med Associates Video-Tracking and Scoring software.

The open field test was performed to evaluate locomotor activity in a rectangular chamber (60 × 60 × 40 cm) made of gray polyvinyl chloride, the central area of which was illuminated by 25-W halogen bulbs (200 cm above the field). Mice were gently placed into the testing chamber for a 5-min recording period, which was monitored using an automated video-tracking system. Images of the paths traveled and time spent in the center in 5 min were both automatically calculated using the DigBehv animal behavior analysis program.

The elevated plus-maze test, which was used to evaluate anxiety-like behaviors, consisted of two opposing open arms (30 × 5 × 0.5 cm) and two opposing enclosed arms (30 × 5 × 15 cm) that were connected by a central platform (5 × 5 cm), forming the shape of a plus sign. All of the measurements were taken in a silent and dimly lit experimental room, to which the mice were acclimatized for at least 30 min before testing. The time spent in the open arms and close arms was recorded over a 5-min test period and automatically calculated using the DigBehv animal behavior analysis program. The maze was cleaned with a solution of 75% ethanol in water between sessions.

### Statistical analyses

To be statistically adequate, we used power = 0.80 to determine the sample size on the basis of the smallest effect we wished to measure to ensure that sample sizes were large enough to detect the effects of interest as an essential part of the study design. For animal experiments, the animals were grouped in a random manner to reduce bias, and during behavioral experiments, animals of different genotypes were staggered so that no two animals from the same group were evaluated consecutively. For all behavioral tests, all the data were autoscored by the EthoVision system in real time so that no human bias was introduced. Thus, no blinding process was involved. For Western blotting, electrophysiological recording and cell counting, the experimenter who conducted the quantification of the genotype of each animal was blinded to the two different genotypes listed as Group A or B. For all the behavioral tests, the data met the assumptions of the tests. The variation estimated within each group was compared, and it was similar between the groups that were being statistically compared. No specific exclusion criteria were applied to the datasets. All of the results are expressed as the means ± s.e.m.s. The statistical analyses were performed using SPSS 30.0 software. Two-tailed Student’s *t* tests were used to compare differences between two groups throughout the study. One-way ANOVA (followed by Bonferroni’s multiple comparisons test) was used for analysis of data from three or more groups. Two-way ANOVA (followed by Bonferroni’s multiple comparisons test) was used to analyze more than two parameters. The significance level for all tests was set at P < 0.05.

## Results

### LFS-induced LTD was age-dependent

**Fig. 1:**
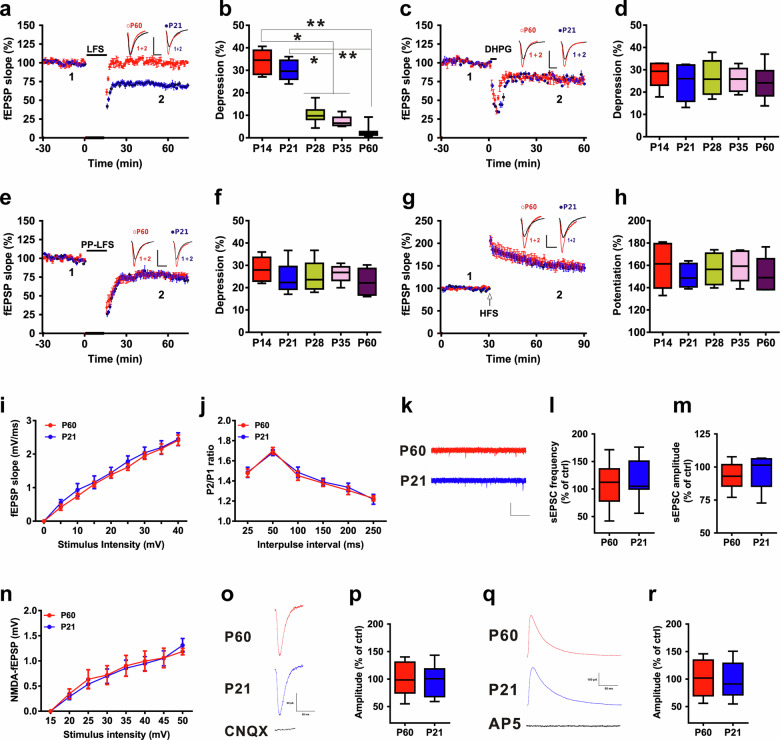
LFS-induced LTD was age dependent. **a**, **b** The developmental profile of LFS-LTDs (n = 8–10 slices/group; one-way ANOVA; *F*_*(*4, 37)_ = 47.807, P < 0.0001). **c**, **d** Summary of the experiments showing the induction of LTD by bath application of DHPG (50 μM) for 5 min to different age groups (n = 6 slices/group; one-way ANOVA; *F*_(4, 24)_ = 21_._674, P = 0.653). **e**, **f** Summary of the experiments showing the induction of LTD by PP-LFS at 1 Hz for 15 min (n = 6 slices/group; one-way ANOVA; *F*_(4, 24)_ = 23_._654, P = 0.428). **g**, **h** The developmental profile of HFS-LTP (n = 6 slices/group; one-way ANOVA; *F*_(4, 24)_ = 17_._873, P = 0.836). Scale bars: 0.5 mV, 5 ms. **i** I–O curves between two age groups (n = 6 slices/group; repeated measures two-way ANOVA, *F*_(1, 90)_ = 17.274, P = 0.867). **j** Differences in the PPF between the two age groups (n = 6 slices/group; two-way repeated-measures ANOVA, *F*_(1, 60)_ = 20.384, P = 0.754). **k**‒**m** The frequency (l, n = 9 slices, two-tailed Student’s *t* test, P = 0.648) and amplitude (m, two-tailed Student’s *t* test, P = 0.734) of sEPSCs in the two age groups. Scale bars: 20 pA, 2 s. **n** NMDAR fEPSP slopes between two age groups (n = 6 slices/group; repeated measures two-way ANOVA, *F*_(1, 80)_ = 21.374, P = 0.703). fEPSPs were recorded in the presence of 20 μM CNQX and 0 nM Mg^2+^. **o**, **p** Differences in evoked NMDA currents between the two age groups (n = 12 cells; two-tailed Student’s *t* test, P = 0.352). Scale bars: 20 pA, 50 ms. **q**, **r** Comparison of evoked AMPA currents between two age groups (n = 12 cells; two-tailed Student’s *t* test, P = 0.721). Scale bars: 100 pA, 50 ms. Data are presented as the mean ± s.e.m. *p < 0.05, **p < 0.01.

LFS reportedly induces NMDAR-dependent LTD at CA3-CA1 hippocampal synapses^5^. We first compared LTD induction in the hippocampal CA1 region in C57BL/6J mice of different ages. Following the demonstration of standard LFS (900 stimuli, 1 Hz)-induced LTD of basal transmission in the hippocampal CA1 region, we discovered that there was a developmental downregulation of LFS-induced LTD: by ~35 days, LFS was less effective at inducing LTD, and by adulthood, LFS did not induce LTD (Fig. 1a, b). Moreover, upon recording LTD in P21 mice, we found that bath application of the N-methyl-D-aspartate (NMDA) receptor (NMDAR) antagonist D-AP5 prevented the induction of LTD (Supplementary Fig. 1a, b), indicating that the induction of LTD at these synapses was dependent on the activation of NMDARs. The application of the alpha-amino-3-hydroxy-5-methyl-4-isoxazolepropionic acid (AMPA) receptor (AMPAR) endocytosis inhibitor GluR2_3Y_ peptide completely abolished the expression of hippocampal LTD (Supplementary Fig. 2a, b). The control inactive peptide GluR2_3A_ failed to affect LTD (Supplementary Fig. 2a, b). This result suggested that AMPAR endocytosis is required for the formation of the LFS-LTDs characterized here.

To more thoroughly examine whether the induction of LTD induced by different stimulation protocols was age dependent, we recorded LTD induced by Group I metabotropic glutamate receptor (mGluR) agonist (S)-3,5-dihydroxyphenylglycine (DHPG) and paired-pulse (PP)-LFS in slices of different ages. We found that bath application of DHPG for 5 min induced a reliable LTD of fEPSPs, with no significant difference in the magnitude of DHPG-induced LTD between different ages (Fig. 1c, d). Moreover, unlike in LFS-LTD, there was no age-related loss of LTD induced by PP-LFS (Fig. 1e, f). These results indicate that the effects of LFS-LTD but not those of DHPG-LTD or PP-LFS-LTD are age dependent. Additionally, NMDA-induced LTD was similar among the age groups (Supplementary Fig. 3a, b). There was no significant difference in LTP induction between the different age groups (Fig. 1g, h). We selected P21 and P60 as representative time points for our next study. Thus, LFS is capable of inducing LTD in slices of young (P21) but not adult hippocampus (P60) (Fig. 1a, b).

### No difference in glutamatergic receptor-mediated synaptic responses between ages

The induction of LTD in the hippocampal CA1 region is predominantly mediated by glutamatergic synaptic transmission^1,39,40^. To investigate the mechanism underlying differences in LTD induction between ages, we first measured fEPSPs at the SC-CA1 synapses in P21 and P60 mouse slices. However, we did not observe any changes in basal synaptic transmission in terms of I‒O curves (Fig. 1i) or in presynaptic release in paired-pulse facilitation (PPF) (Fig. 1j) between the two groups of mice. Moreover, there was no difference in either the frequency or amplitude of spontaneous excitatory postsynaptic currents (sEPSCs) (Fig. 1k–m). These results suggest that AMPAR-mediated synaptic transmission may not be involved in the difference in LTD induction between ages. To determine whether NMDAR-mediated responses were involved, fEPSPs were treated with 20 μM 6-cyano-7-nitroquinoxaline-2,3-dione (CNQX) to block AMPAR and Mg^2+^-free buffer to release the NMDAR block. Interestingly, we found no difference in the slopes of NMDAR fEPSPs because the I‒O curves between the two groups completely overlapped in the above two treatment groups (Fig. 1n), suggesting that NMDAR-mediated synaptic transmission may also not be involved in the difference in LTD induction between ages. To further confirm this finding, we measured AMPA- and NMDA-mediated EPSCs in pyramidal neurons in a whole-cell configuration. We found that AMPAR and NMDAR EPSCs did not change with age, which is in agreement with the findings of previous studies of fEPSPs (Fig. 1o–r).

Together, these observations demonstrate that the downregulation of LTD in adult mouse hippocampal slices is not due to a loss of glutamatergic synaptic transmission.

### An inverse correlation between the 5-HT_1A_ receptor level and the magnitude of LFS-LTD

To characterize the molecular mechanisms underlying the downregulation of LTD, we analyzed the levels of development-related factors that regulate synaptic plasticity in the hippocampus between the two age groups. We found that the monoamine and amino acid concentrations assessed by microdialysis did not change with age (Supplementary Fig. 4a, b). Additionally, the mRNA levels of neurotrophic factors and neuregulins were largely unaltered in the hippocampus (Supplementary Fig. 4c, d).

**Fig. 2:**
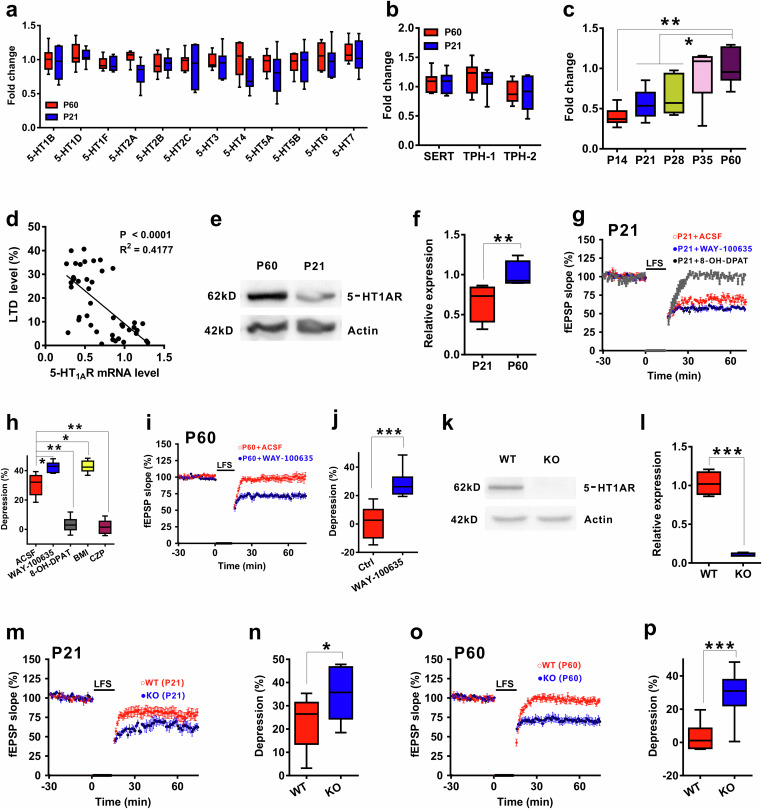
5-HT_1A_ receptor-mediated age-dependent LFS-LTD. **a** Q-PCR measurements of 5-HT receptor profiles in the hippocampal CA1 region from two age groups of mice (n = 7 mice/group; two-tailed Student’s *t* test; 5-HT_1B_, *t*_(12)_ = 1.214, P = 0.186; 5-HT_1D_, *t*_(12)_ = -0.826, P = 0.523; 5-HT_1F_, *t*_(12)_ = -1.093, P = 0.274; 5-HT_2A_, *t*_(12)_ = 1.245, P = 0.385; 5-HT_2B_, *t*_(12)_ = 0.835, P = 0.538; 5-HT_2C_, *t*_(12)_ = 1.374, P = 0.674; 5-HT_3_, *t*_(12)_ = 0.937, P = 0.183; 5-HT_4_, *t*_(12)_ = 1.468, P = 0.109; 5-HT_5A_, *t*_(12)_ = 1.235, P = 0.201; 5-HT_5B_, *t*_(12)_ = 0.246, P = 0.483; 5-HT_6_, *t*_(12)_ = 1.104, P = 0.386; 5-HT_7_, *t*_(12)_ = 0.274, P = 0.208). **b** Q-PCR measurements of 5-HT transporters in CA1 from 21 and 60 day old mice (n = 7 mice/group; two-tailed Student’s *t* test; SERT, *t*_(12)_ = 0.209, P = 0.863; TPH-1, *t*_(12)_ = -0.283, P = 0.736; TPH-2, *t*_(12)_ = -1.832, P = 0.735). **c** Hippocampal 5-HT_1A_R levels at different time points (n = 8–10/group; one-way ANOVA; *F*_(4, 37)_ = 32.138, P = 0.012). **d** Correlation plot of the mRNA level of 5-HT_1A_R against the magnitude of LTD at the developmental age point (*r*^2^ = 0.4177, P < 0.0001). **e**, **f** Western blots showing differences in 5-HT_1A_R protein levels between the two age groups (n = 4 experiments/group; two-tailed Student’s *t* test, P = 0.006). **g**, **h** The 5-HT_1A_R and GABA_A_R agonists impaired LFS-LTD, while their antagonists facilitated LFS-LTD in P21 mice (n = 6 slices/group; one-way ANOVA, *F*_(4, 25)_ = 47.147, P = 0.043). **i**, **j** The 5-HT_1A_R antagonist reversed the impaired LFS-LTD in P60 mice (n = 6-8 slices/group; two-tailed Student’s *t* test, P < 0.001). **k**, **l** Western blots of 5-HT_1A_R in the hippocampus of 5-HT_1A_ R KO mice and their WT littermates (n = 4 experiments/group; two-tailed Student’s *t* test; P < 0.0001). **m**, **n** LFS-LTD was greater in P21 5-HT_1A_R KO mice (n = 7 slices/group; two-tailed Student’s *t* test; P = 0.036). **o**, **p** LFS-LTD was successfully induced in P60 5-HT_1A_R KO mice (n = 7-10 slices/group; two-tailed Student’s *t* test; P < 0.0001). The data are presented as the means ± s.e.m.s; *p < 0.05; **p < 0.01; ***p < 0.001.

As previously mentioned, 5-HT receptors play an important role in establishing proper neural circuits during the early postnatal period^21,23,41–43^. To explore the interaction between the levels of 5-HT receptors and LTD induction, we profiled the expression of 5-HT receptors and found no significant differences in the levels of 5-HT receptors or transporters in the hippocampus between the two age groups (Fig. 2a, b). Interestingly, the mRNA level of the 5-HT_1A_ receptor in the hippocampus increased gradually with age during development (Fig. 2c). Moreover, a statistically significant inverse correlation was observed between the level of the 5-HT_1A_ receptor and the magnitude of LFS-LTD (Fig. 2d). Western blotting also confirmed an increase in the protein level of the 5-HT_1A_ receptor in P60 mice compared with that in P21 mice (Fig. 2e, f). Moreover, the levels of 5-HT_1A_R, 5-HT_2A_R and 5-HT_6_R in the prefrontal cortex and striatum were not different between the two groups (Supplementary Fig. 5a, b). This finding suggested that increased 5-HT_1A_ receptor expression in the hippocampus may contribute to age-dependent LFS-LTD in mice.

### 5-HT_1A_ receptor mediated age-dependent LFS-induced LTD

Having observed an inverse relationship between the 5-HT_1A_ receptor and LFS-LTD during early postnatal development, we next asked whether the 5-HT_1A_ receptor mediates LFS-LTD in the hippocampal CA1 region of mice of different ages. To test this hypothesis, we first applied the selective 5-HT_1A_ receptor agonist 8-OH-DPAT and found that agonist treatment impaired LFS-induced LTD induction in P21 mouse slices (Fig. 2g, h). Moreover, 8-OH-DPAT treatment did not alter basal synaptic transmission or the I‒O curve (Supplementary Fig. 6a, b). We then used the potent 5-HT_1A_ receptor antagonist WAY-100635 and interestingly found that the antagonist facilitated LFS-LTD in P21 mice (Fig. 2g, h) and rescued impaired LFS-induced LTD in P60 mouse slices (Fig. 2i, j). Moreover, WAY-100635 treatment did not alter basal synaptic transmission or the I‒O curve (Supplementary Fig. 6c, d).

To further verify whether the 5-HT_1A_ receptor is involved in LFS-LTD, we used 5-HT_1A_ receptor knockout (KO) mice. Western blotting verified the deletion of the receptors in the KO mice (Fig. 2k, l). We then recorded fEPSPs in the dendritic region of CA1 and compared LTD induction between hippocampal slices taken from control and KO mice. We found that deletion of the receptor increased the magnitude of the LFS-LTD in P21 mice (Fig. 2m, n). Moreover, LFS-LTD was successfully induced in P60 KO mice (Fig. 2o, p). Knockout of the receptor had no effect on the I‒O curve or LTP (Supplementary Fig. 7), which was consistent with the findings of a previous study^44^. These results provide further evidence that 5-HT_1A_ receptors mediate age-dependent LFS-LTD.

### Astrocytic 5-HT_1A_ receptors predominantly mediate LFS-induced LTD

5-HT_1A_ receptors have been reported to be expressed in serotonergic neurons in the dorsal raphe nucleus as autoreceptors and in other cell types in target areas receiving serotonergic innervation, such as the hippocampus, as heteroreceptors^24,25,45^. To evaluate which cell types containing 5-HT_1A_ receptors in the hippocampus are involved in modulating LFS-LTD, we used adeno-associated virus (AAV)–DIO-5-HT_1A_ short-hairpin RNAs (shRNAs) and injected the virus into the CA1 region in different cell type-Cre mice to knock down the receptors in these cells. We first injected the virus into P60 CamKII-Cre mice to knock down the receptors in pyramidal neurons (Supplementary Fig. 8a–j) and found that knockdown of 5-HT_1A_ receptors in pyramidal neurons in the hippocampus had no effect on impaired LFS-LTD in adult mice (Supplementary Fig. 8k, l). We then knocked down the receptors in GABAergic neurons by injecting the virus into GAD-Cre mice (Supplementary Fig. 9a–j). In adult GAD-Cre mice in which 5-HT_1A_ receptors were knocked down, LFS was still unable to induce LTD (Supplementary Fig. 9k, l). These results indicated that pyramidal or GABAergic neuronal 5-HT_1A_ receptors did not affect age-dependent LFS-LTD. Similarly, when the virus was injected into the hippocampal CA1 region of adult Sert-Cre mice to conditionally knock down the receptors in the serotonergic terminals (Supplementary Fig. 10a–j), LFS-LTD was still induced (Supplementary Fig. 10k, l).

**Fig. 3:**
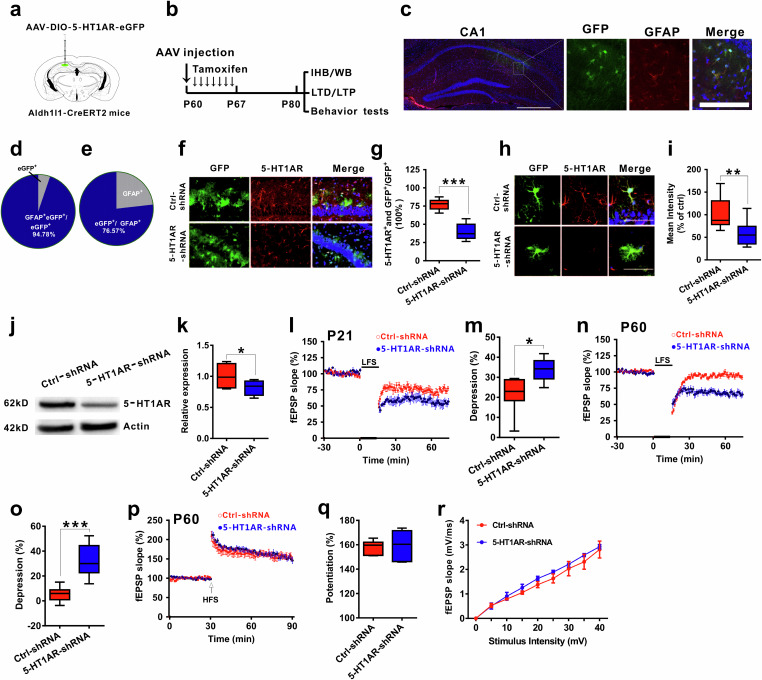
Astrocytic 5-HT_1A_ receptors predominantly modulate LFS-LTD. **a** Schematic of the delivery of AAV-DIO-5-HT_1A_R-eGFP into CA1 in Aldh1l1-CreER^T2^ mice. **b** Schematic of the experiments. **c** Representative location of the 5-HT_1A_R shRNA virus (green) injected into CA1 (left; scale bar, 500 µm) and representative fluorescence images showing that most of the cells infected with AAV-DIO-5-HT_1A_R-eGFP (shRNA) vectors were astrocytes in the CA1 region of aldh1l1-CreER^T2^ mice (right; scale bar, 100 µm). Graphs showing the specificity (**d**, percentage of GFP-positive cells that express GFAP, 94.78 ± 0.38%, n = 865 cells from 10 sections from 4 mice) and efficiency (**e**, percentage of GFAP-positive cells that express GFP, 76.57 ± 2.59%, n = 865 cells from 10 slices from 4 mice) of Cre-mediated recombination in the hippocampal CA1 region of the Aldh1l1-CreER^T2^ transgenic mice infected with AAV-DIO-5-HT_1A_R-eGFP (shRNA) vectors. **f**, **g** Immunofluorescence staining for 5-HT_1A_R (red) and GFP (green) in aldh1l1-CreER^T2^ mice that were injected with pAAV-CAG-DIO-EGFP (control) and shRNA virus. 5-HT_1A_R expression is dramatically reduced in astrocytes in aldh1l1-CreER^T2^ mice that were injected with shRNA virus (Ctrl group: n = 15 cells from four mice; shRNA group: n = 20 cells from five mice; two-tailed Student’s *t* test, P < 0.001). Scale bar: 100 µm. **h** Representative fluorescence images showing the knockdown of 5-HT_1A_R in CA1 astrocytes infected with AAV-DIO-5-HT_1A_R-eGFP (shRNA) vectors in aldh1l1-CreER^T2^ mice. Scale bar: 50 µm. **i** Histogram showing the average fluorescence intensity (red) in CA1 neurons from aldh1l1-CreER^T2^ mice that were injected with pAAV-CAG-DIO-EGFP (control) or shRNA (Ctrl group: n = 15 cells from four mice; shRNA group: n = 20 cells from five mice; two-tailed Student’s *t* test, P = 0.003). The fluorescence intensity of 5-HT_1A_R-positive neurons (red) merged with GFP (green) was plotted using the same imaging conditions for every slice. **j**, **k** Western blots showing 5-HT_1A_R reduction after shRNA-mediated virus injection (n = 4 experiments/group; two-tailed Student’s *t* test, P = 0.031). **l**, **m** Knockdown of astrocytic 5-HT_1A_R facilitated LFS-LTD in P21 mice (n = 6 slices/group; two-tailed Student’s *t* test; P = 0.021). **n**, **o** LFS-LTD was successfully induced in P60 mice in which astrocytic 5-HT_1A_R was knocked down (n = 8–12 slices/group; two-tailed Student’s *t* test; P < 0.0001). **p**, **q** Knockdown of astrocytic 5-HT1ARs had no effect on LTP (n = 6-7 slices/group; two-tailed Student’s *t* test; P = 0.638). **r** I–O curves after knockdown of astrocytic 5-HT_1A_R (n = 6 slices/group; repeated measures two-way ANOVA, *F*_(1, 90)_ = 17.274, P = 0.374). The data are presented as the means ± s.e.m.s; *p < 0.05; ***p < 0.001.

Astrocytes also express 5-HT_1A_ receptors^45–47^. To investigate whether astrocytic 5-HT_1A_ receptors in the hippocampus are critical for modulating LFS-LTD, we used aldehyde dehydrogenase 1 family member L1 (aldh1l1)::CreER^T2^ mice, which were generated in our previous studies^33–35^. We then injected the virus into the CA1 region in aldh1l1-CreER^T2^ adult mice to knock down astrocytic 5-HT_1A_ receptors in the hippocampus induced by one week of intraperitoneal injection of tamoxifen (75 mg/kg; Fig. 3a), and then we recorded the LTD induced by LFS (Fig. 3b). The detection of viral expression indicated by green fluorescence demonstrated that the virus was correctly injected into CA1, and most of the astrocytes were infected with high specificity and efficiency (Fig. 3c–e). In addition, the immunohistochemistry and western blot results verified that the receptor was efficiently knocked down (Fig. 3f–k). Interestingly, we found that knockdown of the receptors in astrocytes facilitated LFS-LTD in P21 mice (Fig. 3l, m) and rescued impaired LFS-LTD in P60 mice (Fig. 3n, o), suggesting a positive role for astrocytic 5-HT_1A_ receptor deletion in age-dependent LFS-LTD and that knockdown of astrocytic 5-HT_1A_ receptors had no effect on the I‒O curve or LTP (Fig. 3p‒r). Interestingly, by purifying astrocytes from mice of different ages via fluorescence-activated cell sorting (FACS) and quantifying 5-HT_1A_ receptors, we found that 5-HT_1A_R expression gradually increased in astrocytes (Supplementary Fig. 11).

### 5-HT_1A_ receptors modulated LFS-induced LTD through enhancing GABAergic transmission

**Fig. 4:**
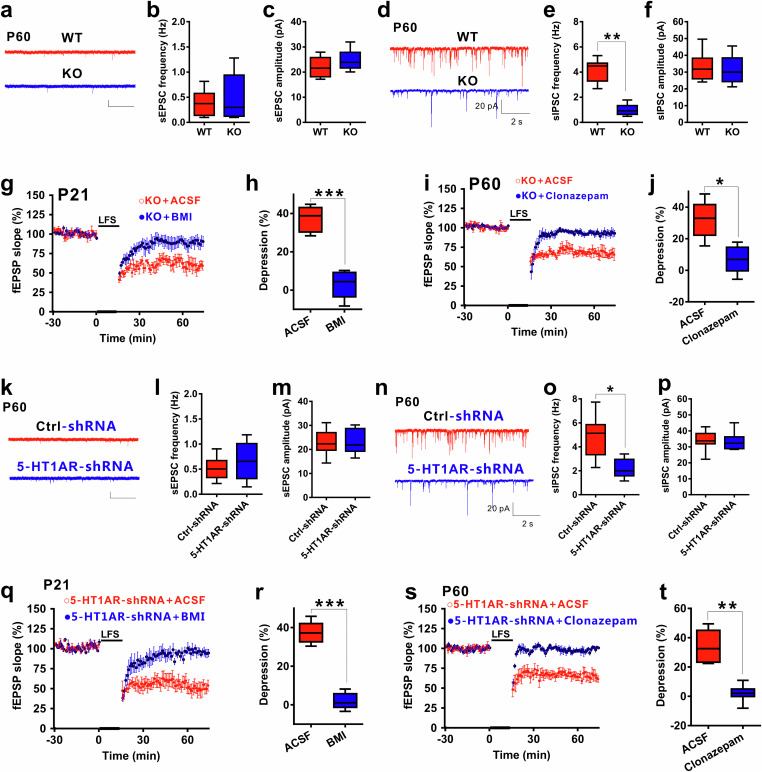
5-HT_1A_ receptors modulated LFS-LTD through decreasing GABAergic transmission. **a** Knockout of 5-HT_1A_R did not affect excitatory glutamatergic transmission (n = 12 cells from 9 slices from 4 mice, two-tailed Student’s t test, for (**b**), P = 0.736; for (**c**), P = 0.683). Scale bars: 20 pA, 2 s. **d** Reduction in inhibitory GABAergic transmission in 5-HT_1A_R KO mice (n = 12 cells from 9 slices from 4 mice, two-tailed Student’s *t* test, for (**e**), P = 0.006; for (**f**), P = 0.362). Scale bars: 20 pA, 2 s. **g**, **h** The GABA_A_ receptor antagonist BMI blocked the effect of knocking out 5-HT_1A_R on P21 LFS-LTD (n = 6 slices from 4 mice, two-tailed Student’s *t* test, P < 0.001). **i**, **j** The GABA_A_ receptor agonist clonazepam blocked the effect of knocking out 5-HT_1A_R on P60 LFS-LTD (n = 6-9 slices from 4 mice, two-tailed Student’s *t* test, P = 0.013). **k** Knockdown of 5-HT_1A_R in astrocytes did not affect excitatory glutamatergic transmission (n = 12 cells from 9 slices from 4 mice, two-tailed Student’s t test, for (**l**), P = 0.374; for (**m**), P = 0.535). Scale bars: 20 pA, 2 s. **n** Reduction in inhibitory GABAergic transmission in astrocytic 5-HT_1A_R-knockdown mice (n = 12 cells from 9 slices from 4 mice, two-tailed Student’s *t* test, for (**o**), P = 0.01; for (**p**), P = 0.417). Scale bars: 20 pA, 2 s. **q**, **r** The GABA_A_ receptor antagonist BMI blocked the effect of knocking down astrocytic 5-HT_1A_R on P21 LFS-LTD (n = 6-9 slices from 4 mice, two-tailed Student’s *t* test, P = 0.008). **s**, **t** The GABA_A_ receptor agonist clonazepam blocked the effect of knocking down astrocytic 5-HT_1A_R on P60 LFS-LTD (n = 6-9 slices from 4 mice, two-tailed Student’s *t* test, P = 0.008). The data are presented as the means ± s.e.m.s; *p < 0.05; **p < 0.01.

We next explored the mechanisms underlying age-dependent LFS-LTD via 5-HT_1A_ receptors. Given that the 5-HT_1A_ receptor is an inhibitory G protein-coupled receptor^24,25^, we hypothesized that increased expression of the 5-HT_1A_ receptor may alter the E/I synaptic balance in CA1 pyramidal neurons, which in turn influences LTD induction. To test this hypothesis, we obtained whole-cell patch-clamp recordings of slices from adult 5-HT_1A_ receptor KO mice and their control littermates. We found that, while sEPSCs were not affected (Fig. 4a–c), knockout of the receptors led to a decrease in the frequency of spontaneous inhibitory postsynaptic currents (sIPSCs) without affecting the amplitude (Fig. 4d–f). Similarly, by comparing sIPSCs and sEPSCs between P21 and P60 mice, we found that the frequency but not the amplitude of sIPSCs in P60 mice was greater than that in P21 mice, without affecting sEPSCs (Supplementary Fig. 12), which was consistent with previous studies^48–50^. Moreover, ex vivo experiments showed that the GABA_A_ receptor antagonist BMI blocked LFS-LTD in P21 5-HT_1A_ receptor KO mice (Fig. 4g, h), while the GABA_A_ receptor agonist clonazepam (5 μM) prevented LFS-LTD in P60 5-HT_1A_ receptor KO mice (Fig. 4i, j). Similar results were also found in astrocytic 5-HT_1A_ receptor knockdown mice (Fig. 4k–t). Moreover, the GABA_A_R agonist impaired LFS-LTD, while an antagonist facilitated LFS-LTD in P21 mice (Fig. 4g, h). The above results support the involvement of GABAergic mechanisms in 5-HT_1A_ receptor-mediated LFS-LTD. However, knockdown of pyramidal or GABAergic neuronal or serotonergic terminal 5-HT_1A_ receptors had no effect on sEPSCs or sIPSCs (Supplementary Figs. 13, 14, 15).

### Hippocampus-dependent fear memory extinction is affected by different treatments

**Fig. 5:**
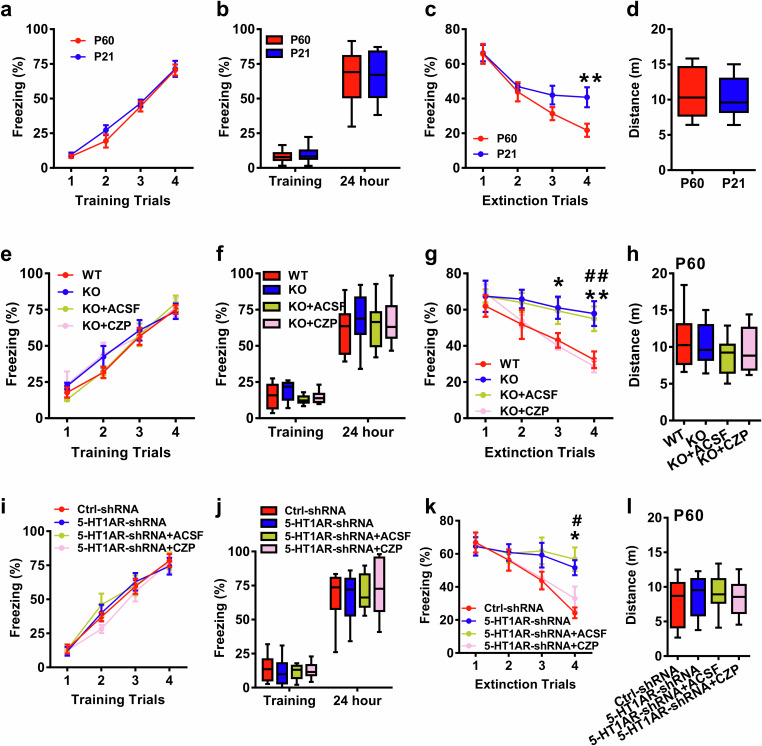
Measurement of the fear memory process in mice subjected to different treatments. **a** Impaired fear memory extinction in P21 mice compared with P60 mice (n = 11-13 mice/group; for a, repeated measures two-way ANOVA, *F*_(1, 88)_ = 19.483, P = 0.735; for (**b**), two-tailed Student’s *t* test, P = 0.638; for (**c**), repeated measures two-way ANOVA, *F*_(1, 88)_ = 38.325, P = 0.043; for (**d**), two-tailed Student’s *t* test, P = 0.825). Knockout of 5-HT_1A_R led to impaired fear memory extinction, which can be rescued by the GABA_A_ receptor agonist clonazepam (CZP) (n = 10 mice/group; for (**e**), repeated measures two-way ANOVA, *F*_(3, 144)_ = 20.853, P = 0.421; for (**f**), one-way ANOVA, *F*_(3, 36)_ = 11.432, P = 0.563; for (**g**), repeated measures two-way ANOVA, *F*_(3, 144)_ = 45.243, P = 0.032, asterisk or hash indicates differences between WT and KO or KO + ACSF and KO + CZP; for (**h**), one-way ANOVA, *F*_(3, 36)_ = 12.417, P = 0.754). Impaired fear memory extinction in astrocytic 5-HT_1A_R knockdown mice can be rescued by the GABA_A_ receptor agonist CZP (n = 10–11 mice/group; for (**i**), repeated measures two-way ANOVA, *F*_(3, 148)_ = 24.468, P = 0.476; for (**j**), one-way ANOVA, *F*_(3, 37)_ = 9.438, P = 0.584; for (**k**), repeated measures two-way ANOVA, *F*_(3, 148)_ = 46.573, P = 0.015; asterisk or hash indicates differences between Ctrl-RNA and 5-HT_1A_R-shRNA or 5-HT_1A_R-shRNA +ACSF and 5-HT_1A_R-shRNA +CZP; for (**l**), one-way ANOVA, *F*_(3, 37)_ = 10.448, P = 0.354). The data are presented as the means ± s.e.m.s; *p < 0.05; **p < 0.01.

LTD is assumed to represent the cellular mechanism underlying fear memory extinction^1,51–55^. It has even been suggested that LTD may represent a synaptic mechanism facilitating selective manipulation of an established memory while retaining the capacity to form new memories^56^. We next employed the contextual fear conditioning test^57^ to investigate whether hippocampal-dependent fear memory extinction was also differentially affected. In P21 and P60 mice, while learning and memory processes were similar, fear memory extinction was slower in P21 group mice than in P60 group mice (Fig. 5a–c). In P21 mice, the selective 5-HT_1A_ receptor agonist 8-OH-DPAT rescued impaired fear memory extinction compared with that in the saline control group (Supplementary Fig. 16). In contrast, treatment with the potent 5-HT_1A_ receptor antagonist WAY-100635 (Supplementary Fig. 17) or knockout of the 5-HT_1A_ receptor impaired fear memory extinction in P60 mice, which was reversed by the GABA_A_ receptor agonist CZP (Fig. 5e–g). This was also the case for the conditional knockdown of the astrocytic 5-HT_1A_ receptor (Fig. 5i–k). There were no obvious differences in locomotor activity under these treatments (Fig. 5d, h, l). As there is a clear correlation between the 5-HT_1A_ receptor and anxiety^58,59^, we also tested anxiety-related behaviors and found that deletion of the astrocytic 5-HT_1A_ receptor induced anxiety-like behaviors both in the open field test and the elevated plus maze test (Supplementary Fig. 18). However, knockdown of pyramidal or GABAergic neuronal or serotonergic terminal 5-HT_1A_ receptors did not affect anxiety-like behaviors (Supplementary Figs. 19, 20, 21). These results support our findings on behaviors and diseases related to LTD.

## Discussion

The most striking feature of LTD is that it is difficult to elicit reliably with the typical LFS in slices from adult hippocampus^10–16^, which makes it difficult to elucidate its role in learning and memory processes. In the present study, we investigated the role of the 5-HT_1A_ receptor in the age-related decrease in the magnitude of LFS-LTD at Schaffer collateral-CA1 synapses. Our results revealed a significant inverse correlation between the expression level of the 5-HT_1A_ receptor and the magnitude of LFS-induced LTD in the hippocampal CA1 region during development. Moreover, 5-HT_1A_ receptor disruption restored the ability of LFS to induce LTD by shifting the E/I synaptic balance toward greater excitation. The expression of the 5-HT_1A_ receptor on astrocytes but not on pyramidal or GABAergic neurons or serotonergic terminals mediated its effect on LFS-LTD. Consistent with this observation, the above treatments also had different effects on fear memory extinction behaviors and anxiety-like behaviors.

Decades of work have consistently reported that LFS becomes less effective at inducing LTD with increasing age^10–16^. Rather than a simple loss of LTD induction ability, previous studies have provided evidence that the intrinsic capacity for LTD is intact in the adult hippocampus but obscured by maturation processes. For example, in vitro manipulations of the extracellular Ca^2+^/Mg^2+^ ratio, as well as the levels of GABA and CaMKII, can result in LTD induction in adult slices^5,10,60–63^. Because of the enhancement of LTD induction by GABA_A_ receptor antagonists in slices from mature animals, it has been suggested that developmental differences in LTD induction may result from the maturation of GABAergic inhibition, which in turn perturbs NMDAR function^61^. Nevertheless, age-dependent changes in glutamate receptors in the hippocampus have also been reported^64^, but we did not observe a difference in glutamatergic receptor-mediated synaptic responses between P21 and P60. This may be due to fact that the neuronal network of the rodent hippocampus is an area that is known to develop into an adult-like state over the course of the first two postnatal weeks. During this period, there is a clear sequence of synapse formation in hippocampal neurons that involves the formation of glutamatergic synapses^64^. Therefore, we did not find a difference between these two ages. Our study extends this notion by showing, for the first time, that 5-HT_1A_ receptor disruption, resulting in a decrease in the level of GABAergic transmission and thereby shifting in the E/I synaptic balance toward greater excitation, allowed for the induction of LFS-LTD in slices from adult mice, further confirming that adult CA1 synapses have the cellular machinery for LFS-LTD. More importantly, we have shown that the enhancement of GABA_A_ receptor function abrogates LTD induction in 5-HT_1A_ receptor disruption slices from adult mice, strongly emphasizing GABAergic inhibition as a molecular brake which limits LTD induction at adult CA1 synapses. These results indicate that 5-HT_1A_ receptor disruption results in a reduction in GABAergic inhibition during LFS, thereby facilitating LFS-LTD induction in adult mice.

Hippocampal LTD can be experimentally induced by several different types of electrical and pharmacological stimulation protocols. LFS-LTD was induced by LFS consisting of 900 pulses delivered at 1 Hz^5^. PP-LFS-LTD consisted of 900 paired pulses at 1 Hz and was repeated two times 10 min apart. In addition, NMDA-LTD and DHPG-LTD were induced by NMDA and DHPG treatment, respectively. In addition to LFS-LTDs, several other forms of LTD have been defined by differences in their induction mechanisms^2^. Our data align with the expectation that chemically induced LTD via direct application of NMDA or DHPG is still possible in slices from adult animals. Although chemically induced LTD did not show age dependency and was readily induced in slices from adult mice, we cannot exclude the possibility that a developmental switch in the synaptic mechanisms of LTD exists to accommodate the age-dependent changes in synaptic properties. Indeed, there is evidence that the synaptic mechanisms and protein synthesis of CA1 mGluR-LTD change with developmental age^65^. Results demonstrated that the magnitude of DHPG-LTD was greater in aged male rats (22–26 months) than in young adult rats (5–8 months); however, in our study, we compared DHPG-LTD between P21 and P60 mice and found no difference. The finding that 5-HT_1A_ receptor disruption had no effect on the magnitude of NMDA-, DHPG-, or PP-LFS-induced LTD indicates that 5-HT_1A_ receptors specifically restrict LFS-LTD at Schaffer collateral-CA1 synapses. One possible explanation for the different effects of 5-HT_1A_ receptors on distinct forms of LTD is likely the differential influence of GABAergic inhibition on their induction, as blockade of GABAergic synaptic transmission does not affect DHPG- and PP-LFS-induced LTD at Schaffer collateral-CA1 synapses^66,67^.

5-HT is a biogenic amine that acts as a neurotransmitter and neuromodulator. Within the 5-HT system, signaling through inhibitory 5-HT_1A_ is required for the normal development of circuits that subserve brain function in mice^20–23^. The evidence from transgenic approaches suggests that normal expression of 5-HT_1A_ receptors is required in the 2nd and 3rd weeks of life for the emergence of normal anxiety^21^. Similar results were observed as a result of pharmacological blockade of 5-HT_1A_ receptors from postnatal Days P0-P21 or from P13-P34^68,69^. Furthermore, pharmacological and genetic mouse models also suggest that the receptor is dispensable for normal anxiety-like behavior in adult animals^70^. Thus, the evidence available to date indicates that once formed, the circuits are either sufficiently stable to withstand the loss of 5-HT_1A_ receptors or that 5-HT_1A_ receptors play a different role in adulthood than they do in development. Nevertheless, little is known about the function of 5-HT_1A_ receptors in regulating age-dependent LFS-LTD and its related mental disorders. Indeed, numerous mental processes undergo a shift in the structures that support their function during development. For example, fear extinction during early development depends primarily on the amygdala, whereas joint roles for the amygdala, medial prefrontal cortex and hippocampus emerge later^71^. Thus, 5-HT_1A_ receptors may play a role in age-dependent LFS-induced LTD. Here, we show, for the first time, that increased 5-HT_1A_ receptor expression is inversely correlated with a decrease in LFS-LTD during development. The results from transgenic and pharmacological studies also provide additional evidence that the 5-HT_1A_ receptor modulates LFS-LTD. Altogether, these findings revealed that the 5-HT_1A_ receptor is a previously unrecognized negative regulator of age-dependent LTD induction in the hippocampal CA1 region. The 5-HT_1A_ receptor is predominantly a somatodendritic autoreceptor in the neurons of the raphé nucleus that regulates the amount of 5-HT released and therefore serotonergic activity in different projection areas^24,25^. Additionally, 5-HT_1A_ receptor expression has been described in forebrain areas, including the hippocampus, that are involved in learning, control of emotions, memory and fear-related information^24,25^. Our results support an active role for the astrocytic 5-HT_1A_ receptor in modulating age-dependent LFS-LTD, which also supports the positive role of astrocytes in brain function. Astrocytes are abundant glial cells that mediate synaptic plasticity through their intracellular Ca^2+^ signals^72^. In our previous study, we found that temporal integration of Ca^2+^ transients in astrocytes by repeated HFS is essential for late-phase LTP^35^. Future studies will provide some evidence to support the contribution of LFS-induced Ca^2+^ integration in astrocytes to LTD induction. Astrocytes can release a variety of synaptic transmitters and modulators, including but not limited to glutamate, D-serine, ATP/adenosine, GABA and lactate, through calcium-dependent and calcium-independent signaling pathways^73^. Whether the gliotransmitters released from astrocytes activate the 5-HT_1A_ receptor and how they mediate GABA transmission will be investigated in future studies.

P21 and P60 mice were subjected to hippocampus-dependent fear memory extinction. The results indicated that P21 mice exhibited slower fear memory extinction that was accompanied by intact LFS-LTD, while in P60 mice, normal fear memory extinction accompanied impaired LFS-LTD. Although we are unsure about the molecular mechanisms underlying LTD abnormalities in P60 mice, it is possible that consistent downregulation of some of the plasticity-related neuronal genes observed in P60 mouse brains may underlie such impairment. For example, LTD is regulated by AMPA receptor trafficking^74,75^. As we observed that the AMPAR endocytosis inhibitor GluR2_3Y_ peptide completely abolished the expression of hippocampal LTD (Supplementary Fig. 2a, b), it is tempting to speculate that LTD abnormalities in P60 mice may be at least partially a result of impairment in AMPA receptor trafficking^76^. Interestingly, disruption of AMPA receptor endocytosis has also been shown to impair extinction, but not acquisition, of learned fear^56^. A number of previous studies have demonstrated a connection between LTD and memory extinction^1,51–55^. It has even been proposed that LTD may represent a synaptic mechanism facilitating selective manipulation of an established memory while retaining the capacity to form new memories^56^. Therefore, our study provides a valuable strategy for further investigation of the precise mechanistic connection between LTD and memory extinction.

In summary, we demonstrated a novel mechanism underlying the age-related decrease in LFS-LTD. The results of this study suggest an unidentified and important role for the astrocytic 5-HT_1A_ receptor in restricting LFS-LTD. Our results also revealed that 5-HT_1A_ receptor blockade restores the ability of LFS to induce LTD at adult synapses by shifting the E/I synaptic balance. These findings further increase our understanding of the mechanisms by which 5-HT_1A_ receptors control synaptic plasticity in the hippocampus. Thus, to our knowledge, our results are the first to demonstrate the critical contribution of the astrocytic 5-HT_1A_ receptor in modulating age-dependent LFS-LTD and fear memory extinction, which may provide alternative approaches for treating a diverse range of disorders related to LTD.

## Supplementary information

Supplementary Fig. 1-21
