# Supplementary material for: Astrocytic 5-HT1A receptor mediates age-dependent hippocampal LTD and fear memory extinction in male mice
Source: Exp Mol Med. 2024 Aug 1;56(8):1763–75. doi: 10.1038/s12276-024-01285-0 (PMC11371825; doi:10.1038/s12276-024-01285-0)
Supplement: Supplementary file 1 — Supplementary Fig. 1-21 [file 12276_2024_1285_MOESM1_ESM.pdf]

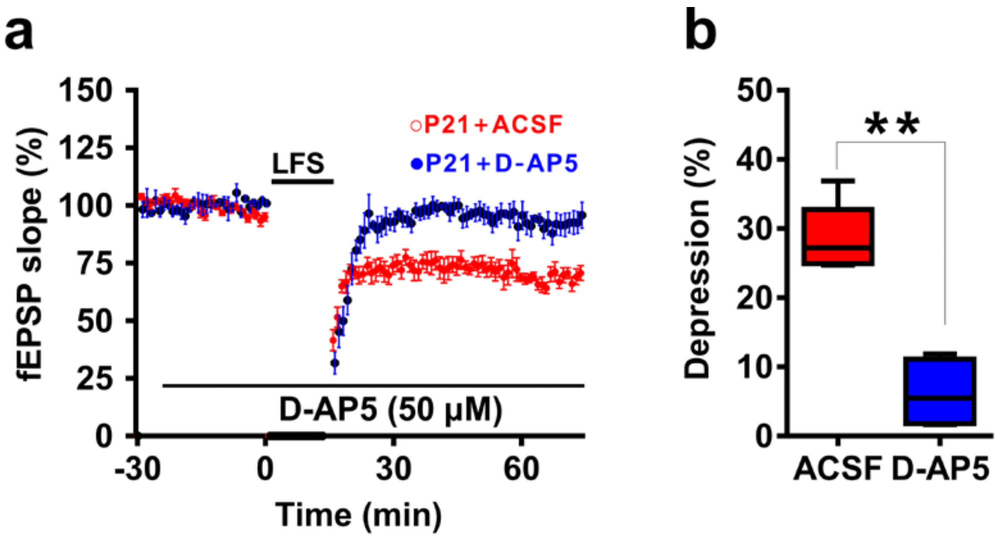

Supplementary Fig. 1

NMDAR-dependence of LTD in P21 mice.

(**a, b**) LFS-induced LTD in P21 mice is sensitive to NMDAR inhibition (n=5-6 slices from 3 mice /group, two-tailed Student's t-test, P=0.008).

Data were presented as mean ± s.e.m. **p < 0.01.

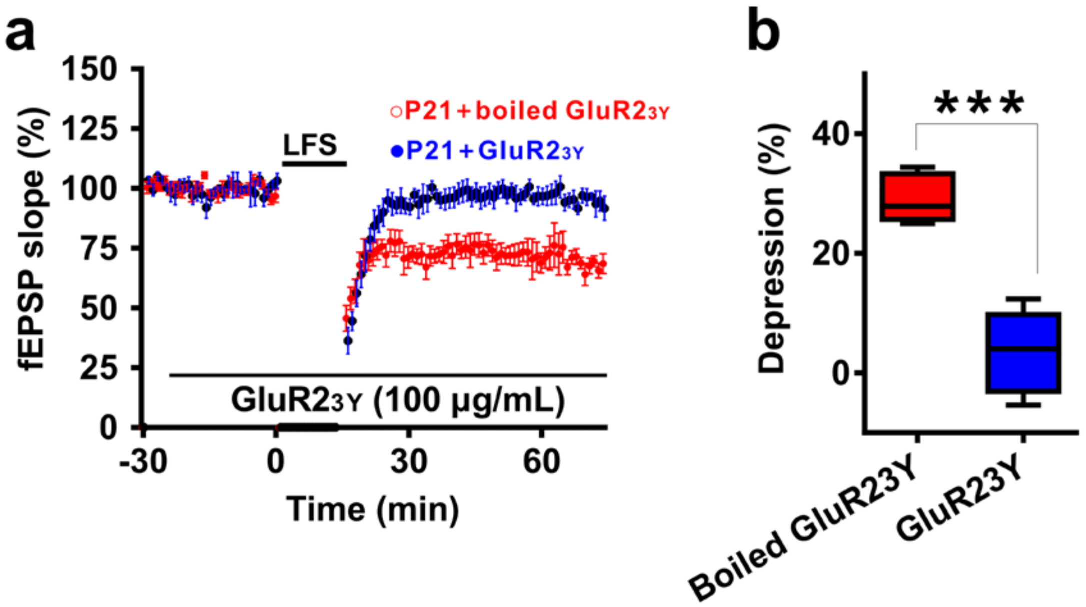

Supplementary Fig. 2

AMPAR endocytosis-dependence of LTD in P21 mice.

(**a, b**) LFS-induced LTD in P21 mice is sensitive to AMPAR endocytosis inhibition (n=5 slices from 3 mice /group, two-tailed Student's t-test, P<0.001).

Data were presented as mean ± s.e.m. ***p < 0.001.

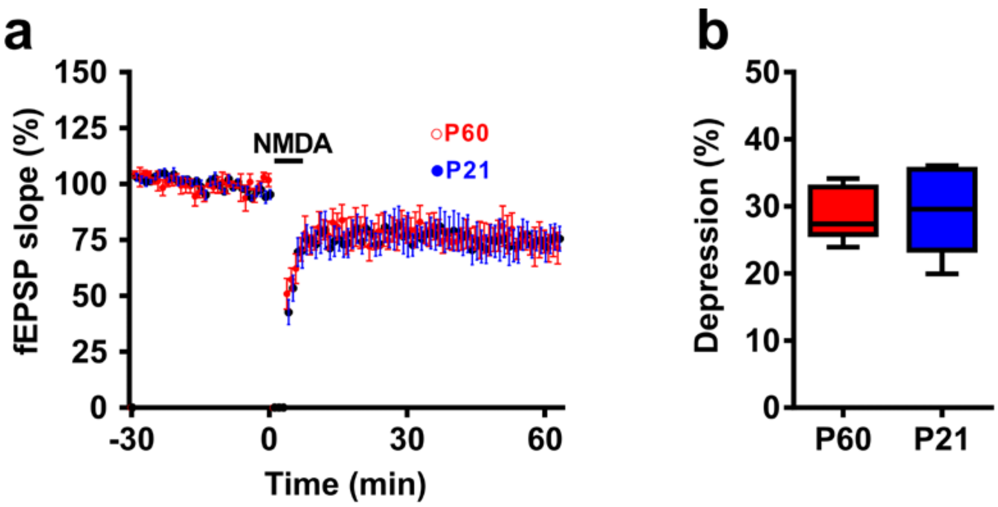

Supplementary Fig. 3

NMDA-induced LTD in P60 and P21 mice.

(**a, b**) Summary of experiments showing the induction of LTD by bath application of NMDA (15 μM) for 3 min in P60 and P21 mice slices (n=6 slices from 3 mice /group, two-tailed Student's t-test, P=0.381).

Data were presented as mean ± s.e.m. ***p < 0.001.

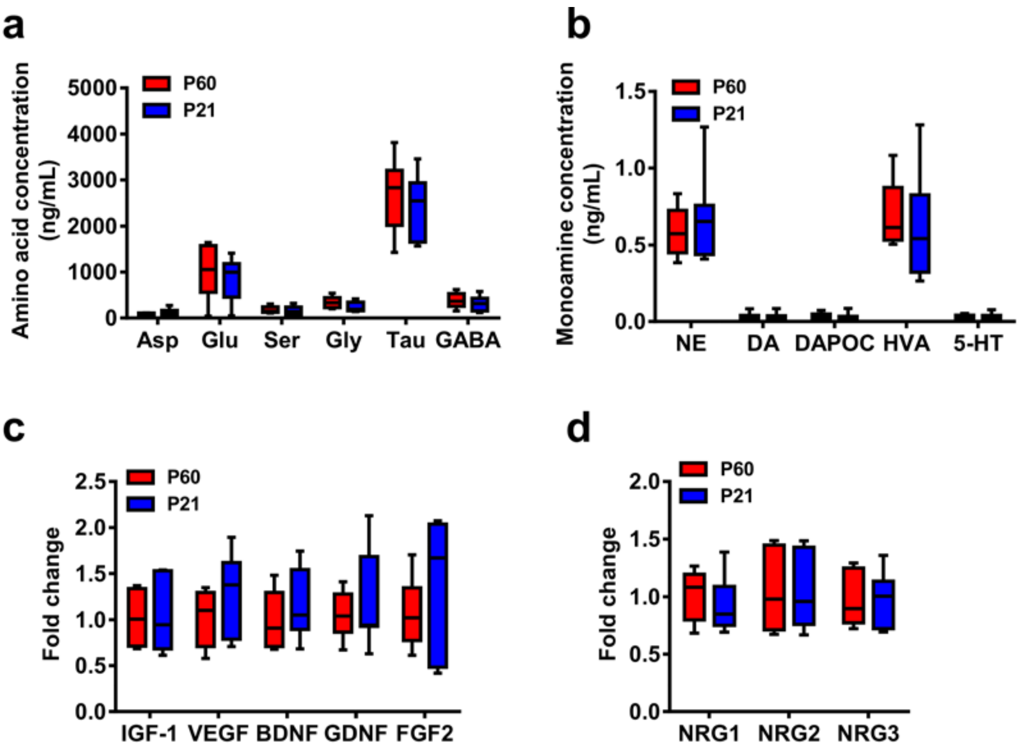

Supplementary Fig. 4

Measurements of amino acids, monoamines and trophic factors between two age groups.

(a) Microdialysis measurements of amino acids concentration in the hippocampal CA1 region from two age groups mice (n = 6 mice/group; two-tailed Student's t-test; Asp: aspartic acid, $t_{(10)} = -2.243$, P = 0.465; Glu: glutamate, $t_{(10)} = 0.314$, P = 0.334; Ser: serine, $t_{(10)} = 0.247$, P = 0.259; Gly: glycine, $t_{(10)} = 1.137$, P = 0.316; Tau: taurine, $t_{(10)} = 1.095$, P = 0.391; GABA: γ-aminobutyric acid, $t_{(10)} = 1.097$, P = 0.097).

(b) Microdialysis measurements of monoamines concentration in the hippocampal CA1 region from two age groups mice (n = 6 mice/group; two-tailed Student's t-test; NE: norepinephrine, $t_{(10)} = -0.167$, P = 0.258; DA: dopamine, $t_{(10)} = 1.147$, P = 0.352; DAPOC: 3,4-Dihydroxybenzoic acid, a metabolin of DA, $t_{(10)} = 0.876$, P = 0.479; HVA: 4-hydroxy-3-methoxyphenylacetic, another metabolin of DA, $t_{(10)} = 1.094$, P = 0.136; 5-HT: serotonin, $t_{(10)} = 1.684$, P = 0.587).

(c) Q-PCR measurements of trophic factors level in the hippocampal CA1 region from two age groups mice (n = 5 mice/group; two-tailed Student's t-test; IGF-1: insulin-like growth factor 1, $t_{(8)} = -0.587$, P = 0.531; VEGF: vascular endothelial growth factor, $t_{(8)} = -0.575$, P

= 0.556; BDNF: brain-derived neurotrophic factor, $t_{(8)}$ = -1.314, P = 0.468; GDNF: glial cell line-derived neurotrophic factor, $t_{(8)}$ = -1.358, P = 0.531; FGF-2: fibroblast growth factor 2, $t_{(8)}$ = -0.368, P = 0.632).

(d) Measurement of NRG subtype mRNA levels in the hippocampal CA1 region from two age groups mice (n = 6 mice/group; two-tailed Student's t-test; NRG-1: $t_{(10)}$ = 1.256, P = 0.354; NRG-2: $t_{(10)}$ = -0.875, P = 0.461; NRG-3: $t_{(10)}$ = 0.358, P = 0.621).

Data were presented as mean ± s.e.m.

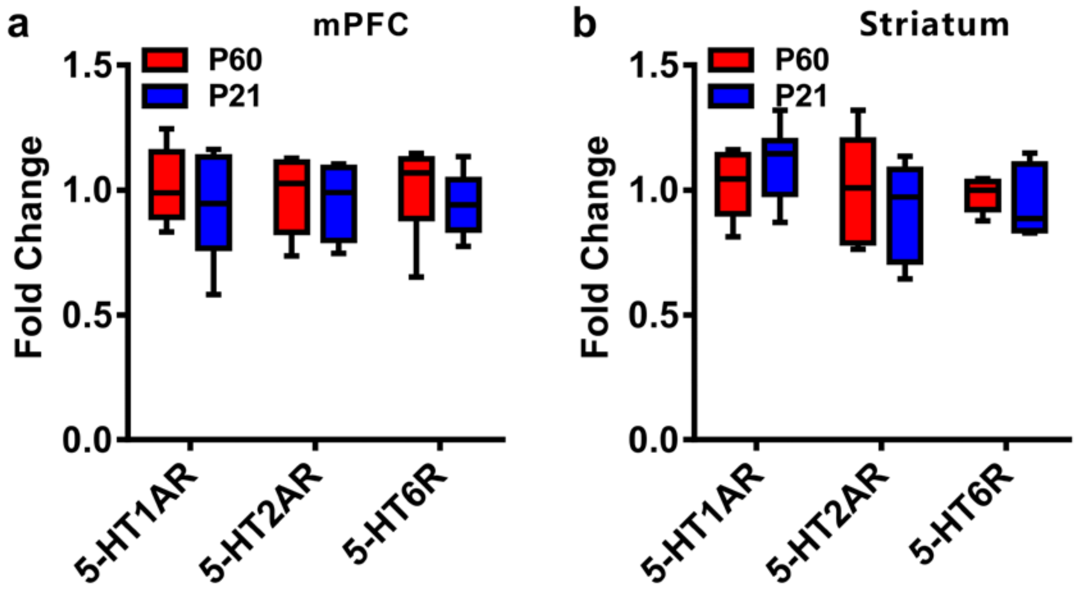

Supplementary Fig. 5

The expression levels of 5-HT$_{1A}$R, 5-HT$_{2A}$R and 5-HT$_6$R in the mPFC and striatum between two age groups.

(a) Q-PCR measurements of 5-HT$_{1A}$R, 5-HT$_{2A}$R and 5-HT$_6$R in the mPFC from two age group mice (n = 6 mice/group; two-tailed Student's t-test; 5-HT$_{1A}$R, $t_{(10)} = 1.093$, $P = 0.736$; 5-HT$_{2A}$R, $t_{(10)} = 0.364$, $P = 0.937$; 5-HT$_6$R, $t_{(10)} = 1.634$, $P = 0.734$).

(b) Q-PCR measurements of 5-HT$_{1A}$R, 5-HT$_{2A}$R and 5-HT$_6$R in the striatum from two age group mice (n = 6 mice/group; two-tailed Student's t-test; 5-HT$_{1A}$R, $t_{(10)} = 1.683$, $P = 0.537$; 5-HT$_{2A}$R, $t_{(10)} = 0.673$, $P = 0.473$; 5-HT$_6$R, $t_{(10)} = 1.732$, $P = 0.384$).

Data were presented as mean ± s.e.m.

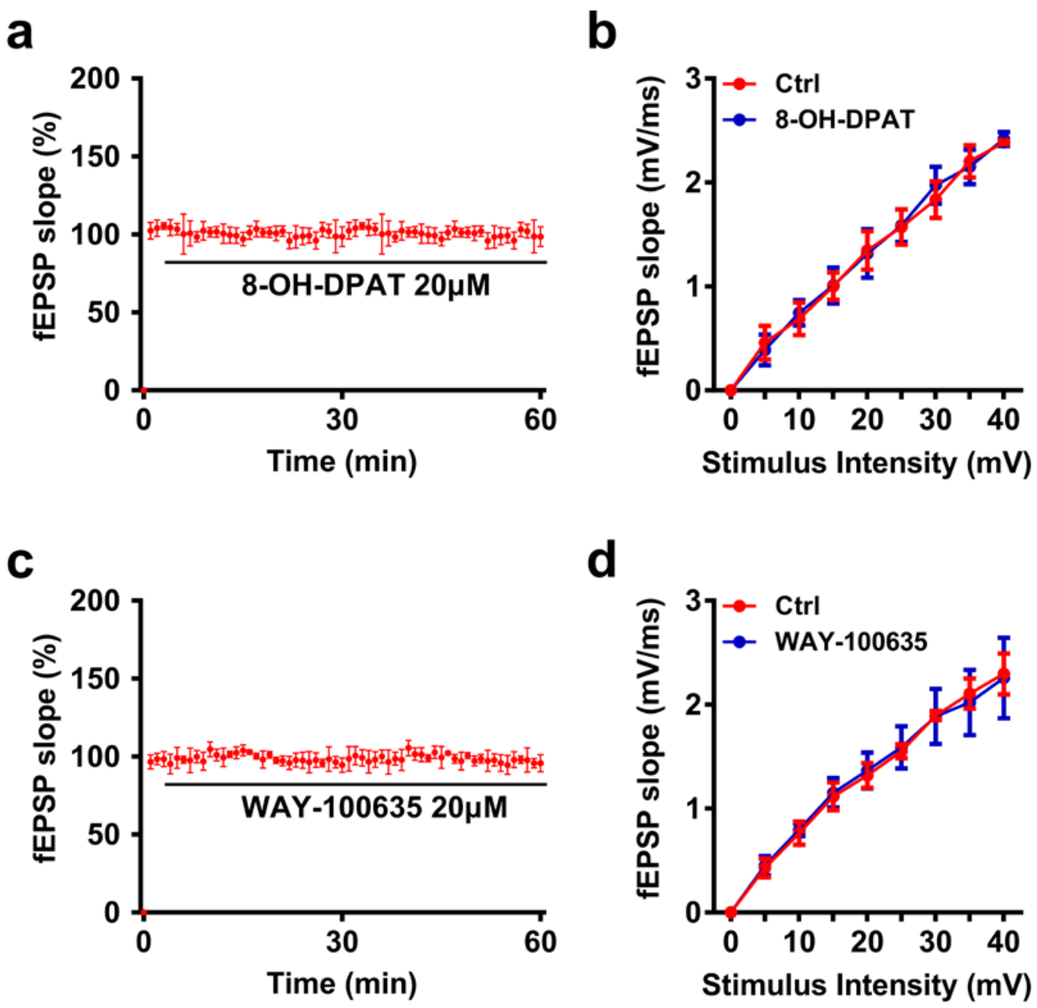

Supplementary Fig. 6

Treatment of 5-HT$_{1A}$R agonist or antagonist had no effect on basal neurotransmission or I-O curve.

(a) Bath application of 8-OH-DPAT after establishing the baseline recording did not affect the fEPSP slope (n = 5 slices).

(b) I–O curves after acute 8-OH-DPAT treatment (n =6 slices/group; repeated measures two-way ANOVA, $F_{(1, 90)}$ = 15.274, P = 0.485).

(c) Bath application of WAY-100635 after establishing the baseline recording did not affect the fEPSP slope (n = 5 slices).

(d) I–O curves after acute WAY-100635 treatment (n =6 slices/group; repeated measures two-way ANOVA, $F_{(1, 90)}$ = 16.683, P = 0.725).

Data were presented as mean ± s.e.m.

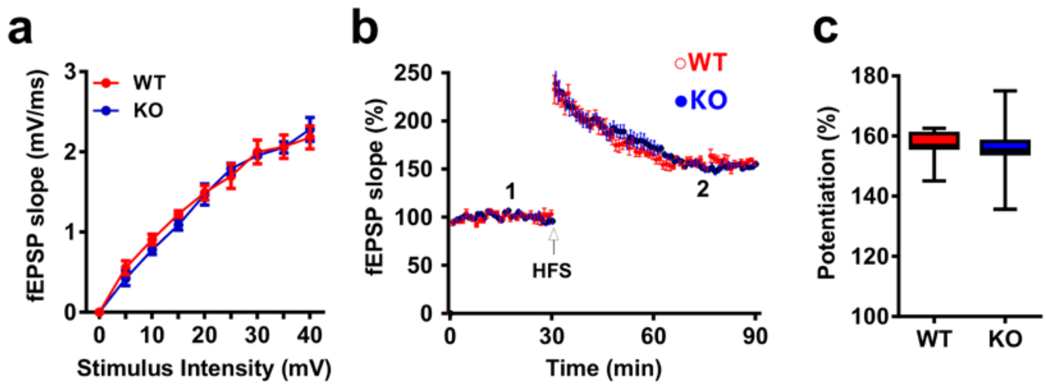

Supplementary Fig. 7

Knockout of 5-HT$_{1A}$R had no effect on I-O curve or LTP.

(a) I–O curves after knockout of 5-HT$_{1A}$R (n =6 slices/group; repeated measures two-way ANOVA, $F_{(1, 90)}$ = 18.623, P = 0.764).

(b, c) LTP induction after knockout of 5-HT$_{1A}$R (n =6 slices/group; two-tailed Student's t-test; P = 0.887).

Data were presented as mean ± s.e.m.

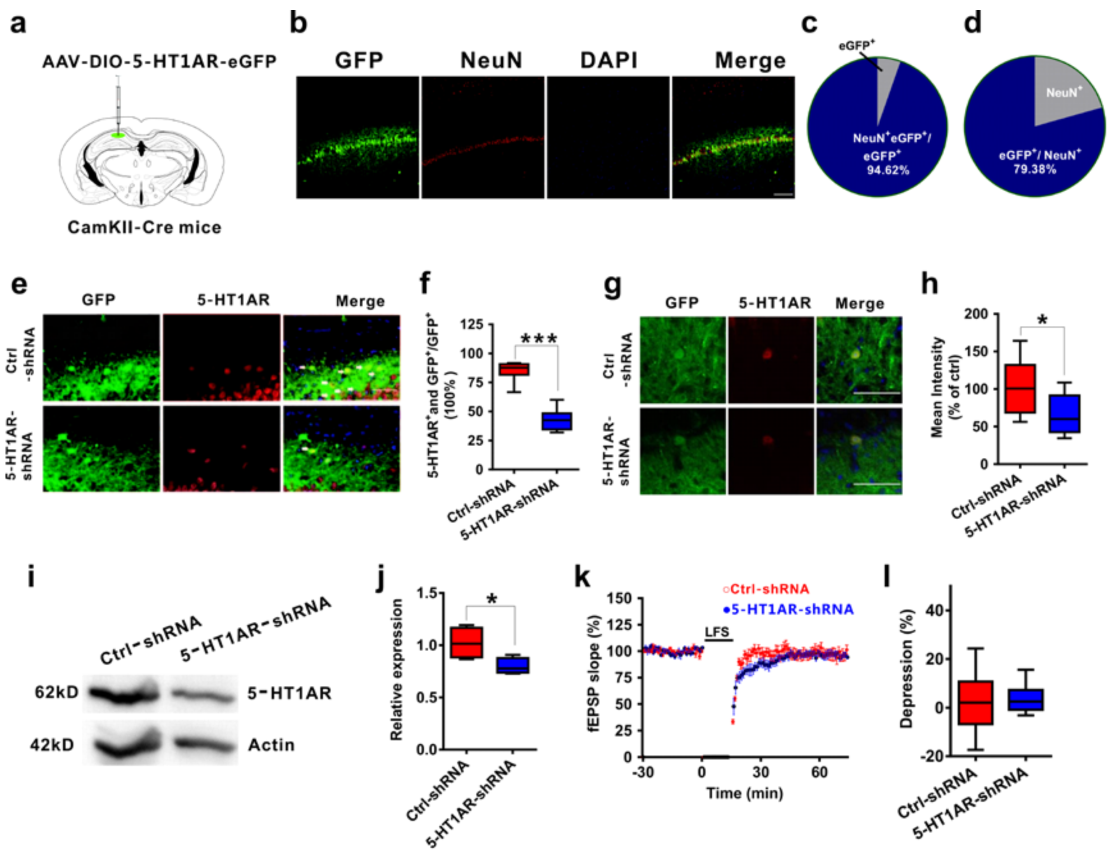

Supplementary Fig. 8

Hippocampal 5-HT1ARs in pyramidal neurons are not necessary for LFS-LTD.

(**a**) Schematic of the delivery of AAV-DIO-5-HT1AR-eGFP into the CA1 in CamKII-Cre mice.

(**b**) Representative fluorescence images showing that most of the cells infected with AAV-DIO-5-HT1AR -eGFP (shRNA) vectors were pyramidal neurons in the CA1 of CamKII-Cre mice. Scale bar, 125 μm.

(**c, d**) Graphs showing the specificity (c, the percentage of GFP positive cells that express NeuN, 94.62±1.26%, n=964 cells from 10 slices from 4 mice) and the efficiency (d, the percentage of GFAP positive cells that express GFP, 79.38±1.34%, n=964 cells from 10 slices from 4 mice) of Cre-mediated recombination in the hippocampal CA1 of the CamKII-Cre transgenic mice infected with AAV-DIO-5-HT1AR -eGFP (shRNA) vectors.

(**e, f**) Immunofluorescence staining to detect 5-HT1AR (red) and GFP (green) in CamKII-Cre mice that were injected with pAAV-CAG-DIO-EGFP (control) and shRNA virus. 5-HT1AR expression is dramatically reduced in pyramidal neurons in CamKII-Cre mice that were injected with shRNA virus (Ctrl group: n =15 cells from four mice; shRNA

group: n = 20 cells from five mice; two-tailed Student's t-test, P < 0.001). Scale bar: 100 µm.

(**g**) Representative fluorescence images showing the knockdown of 5-HT_{1A}R in CA1 pyramidal neurons infected with AAV-DIO-5-HT_{1A}R -eGFP (shRNA) vectors in CamKII-Cre mice. Scale bar: 50 µm.

(**h**) Histogram showing average fluorescence intensity (red) in the CA1 neurons from CamKII-Cre mice that were injected with pAAV-CAG-DIO-EGFP (control) and shRNA virus (Ctrl group: n =15 cells from four mice; shRNA group: n = 20 cells from five mice; two-tailed Student's t-test, P = 0.01). The fluorescence intensity of the 5-HT_{1A}R-positive neurons (red) merged with GFP (green) was plotted using the same imaging conditions for every slice.

(**i, j**) Western blots showing 5-HT_{1A}R reduction after shRNA virus injection (n= 4 experiments/group; two-tailed Student's t-test, P = 0.028).

(**k, l**) LFS-LTD was unsuccessfully induced in P60 mice that were knocked down 5-HT_{1A}Rs in pyramidal neurons (n = 8 slices/group; two-tailed Student's t-test; P=0.463). Data were presented as mean ± s.e.m; *, p < 0.05.

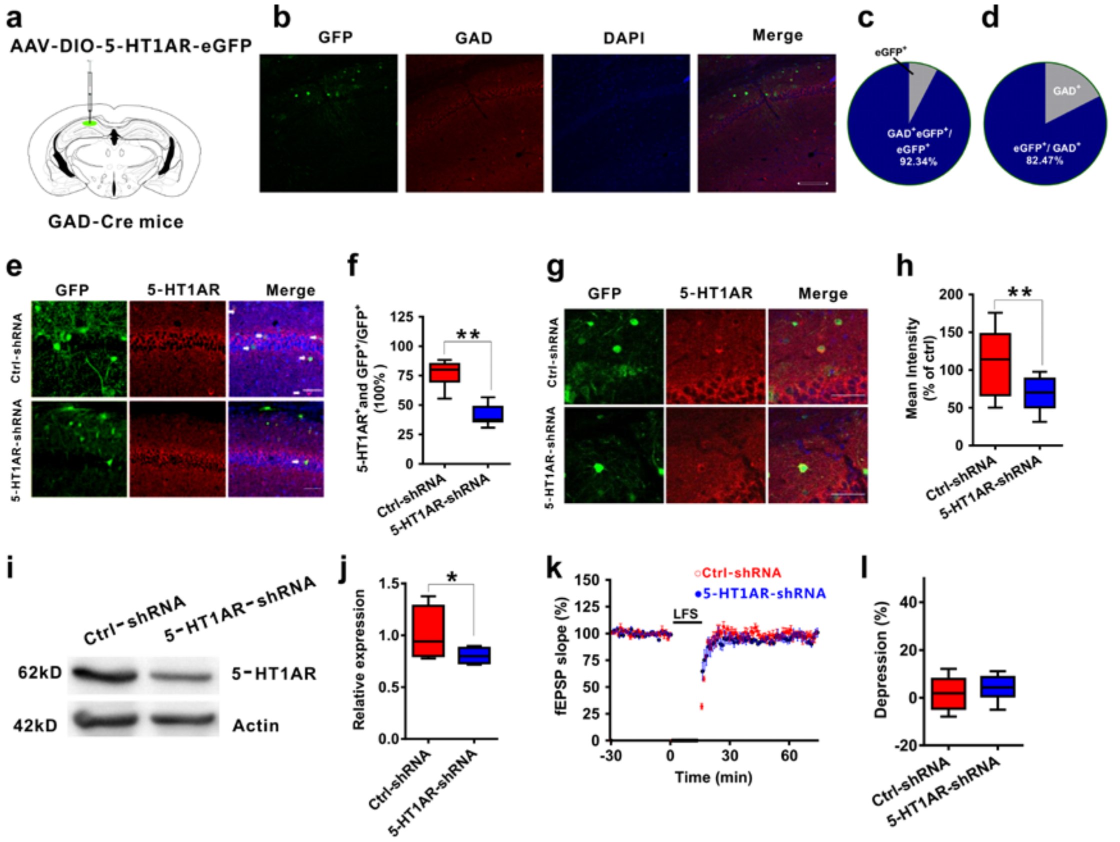

Supplementary Fig. 9

Hippocampal 5-HT$_{1A}$Rs in GABAergic neurons are not necessary for LFS-LTD.

(**a**) Schematic of the delivery of AAV-DIO-5-HT$_{1A}$R-eGFP into the CA1 in GAD-Cre mice.

(**b**) Representative fluorescence images showing that most of the cells infected with AAV-DIO-5-HT$_{1A}$R -eGFP (shRNA) vectors were GABAergic neurons in the CA1 of GAD-Cre mice. Scale bar, 125 µm.

(**c, d**) Graphs showing the specificity (c, the percentage of GFP positive cells that express GAD, 92.34±1.09%, n=578 cells from 10 slices from 4 mice) and the efficiency (d, the percentage of GAD positive cells that express GFP, 82.47±1.68%, n=578 cells from 10 slices from 4 mice) of Cre-mediated recombination in the hippocampal CA1 of the GAD-Cre transgenic mice infected with AAV-DIO-5-HT$_{1A}$R -eGFP (shRNA) vectors.

(**e, f**) Immunofluorescence staining to detect 5-HT$_{1A}$R (red) and GFP (green) in GAD-Cre mice that were injected with pAAV-CAG-DIO-EGFP (control) and shRNA virus. 5-HT$_{1A}$R expression is dramatically reduced in GABAergic neurons in GAD-Cre mice that were injected with shRNA virus (Ctrl group: n =15 cells from four mice; shRNA group: n = 20 cells from five mice; two-tailed Student's t-test, P = 0.002). Scale bar: 100 µm.

(**g**) Representative fluorescence images showing the knockdown of 5-HT_{1A}R in CA1 GABAergic neurons infected with AAV-DIO-5-HT_{1A}R -eGFP (shRNA) vectors in GAD-Cre mice. Scale bar: 50 µm.

(**h**) Histogram showing average fluorescence intensity (red) in the CA1 neurons from GAD-Cre mice that were injected with pAAV-CAG-DIO-EGFP (control) and shRNA virus (Ctrl group: n =15 cells from four mice; shRNA group: n = 20 cells from five mice; two-tailed Student's t-test, P = 0.005). The fluorescence intensity of the 5-HT_{1A}R-positive neurons (red) merged with GFP (green) was plotted using the same imaging conditions for every slice.

(**i, j**) Western blots showing 5-HT_{1A}R reduction after shRNA virus injection (n= 4 experiments/group; two-tailed Student's t-test, P = 0.022).

(**k, l**) LFS-LTD was unsuccessfully induced in P60 mice that were knocked down 5-HT_{1A}Rs in GABAergic neurons (n = 8 slices/group; two-tailed Student's t-test; P=0.358). Data were presented as mean ± s.e.m; *, $p < 0.05$.

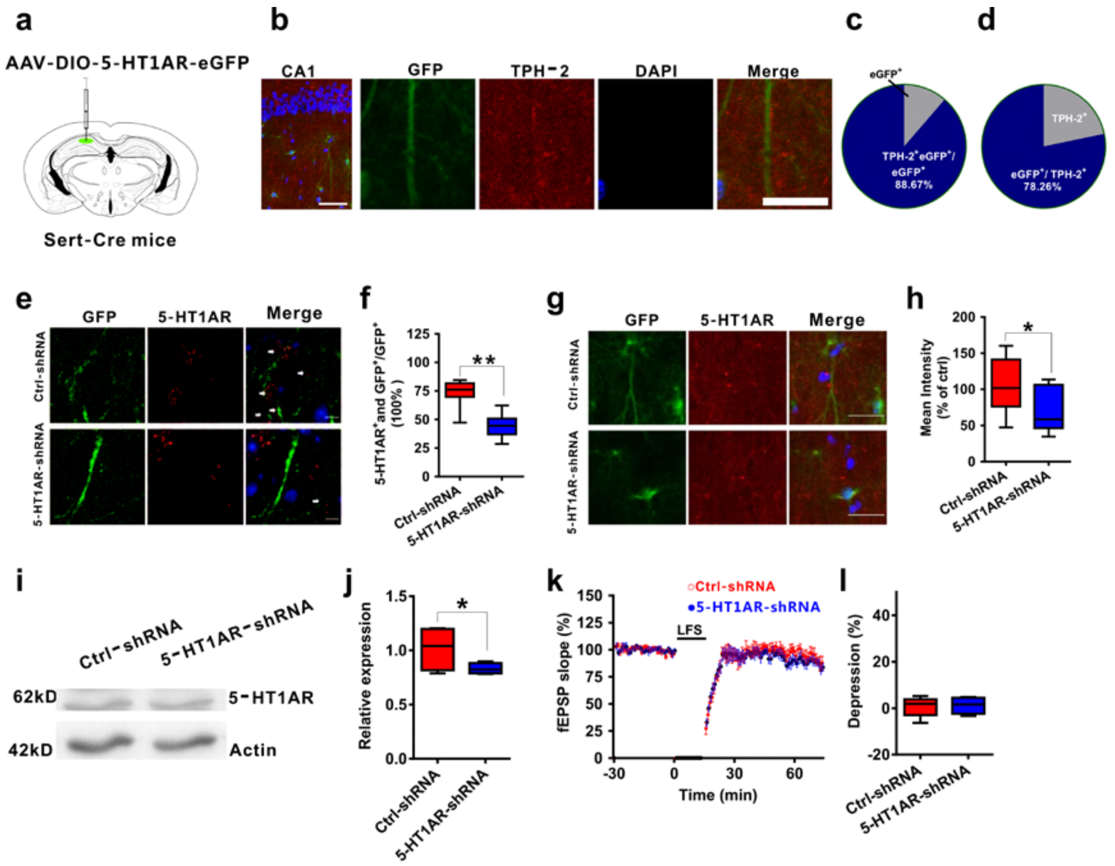

Supplementary Fig. 10

Hippocampal 5-HT$_{1A}$Rs in serotonergic terminals are not necessary for LFS-LTD.

(**a**) Schematic of the delivery of AAV-DIO-5-HT$_{1A}$R-eGFP into the CA1 in sert-Cre mice.

(**b**) Representative fluorescence images showing that most of the cells infected with AAV-DIO-5-HT$_{1A}$R -eGFP (shRNA) vectors were serotonergic terminals in the CA1 of sert-Cre mice. Scale bar, left: 50 µm; right: 20µm.

(**c, d**) Graphs showing the specificity (c, the percentage of GFP positive cells that express TPH-2, 88.67±2.76%, n=832 cells from 10 slices from 4 mice) and the efficiency (d, the percentage of GAD positive cells that express GFP, 78.26±3.45%, n=832 cells from 10 slices from 4 mice) of Cre-mediated recombination in the hippocampal CA1 of the Sert-Cre transgenic mice infected with AAV-DIO-5-HT$_{1A}$R -eGFP (shRNA) vectors.

(**e, f**) Immunofluorescence staining to detect 5-HT$_{1A}$R (red) and GFP (green) in Sert-Cre mice that were injected with pAAV-CAG-DIO-EGFP (control) and shRNA virus. 5-HT$_{1A}$R expression is dramatically reduced in serotonergic terminals in Sert-Cre mice that were injected with shRNA virus (Ctrl group: n =15 cells from four mice; shRNA group: n = 20 cells from five mice; two-tailed Student’s t-test, P = 0.004). Scale bar: 100 µm.

(**g**) Representative fluorescence images showing the knockdown of 5-HT_{1A}R in CA1 serotonergic terminals infected with AAV-DIO-5-HT_{1A}R -eGFP (shRNA) vectors in Sert-Cre mice. Scale bar: 50 µm.

(**h**) Histogram showing average fluorescence intensity (red) in the CA1 neurons from Sert-Cre mice that were injected with pAAV-CAG-DIO-EGFP (control) and shRNA virus (Ctrl group: n =15 cells from four mice; shRNA group: n = 20 cells from five mice; two-tailed Student's t-test, P = 0.02). The fluorescence intensity of the 5-HT_{1A}R-positive neurons (red) merged with GFP (green) was plotted using the same imaging conditions for every slice.

(**i, j**) Western blots showing 5-HT_{1A}R reduction after shRNA virus injection (n= 4 experiments/group; two-tailed Student's t-test, P = 0.044).

(**k, l**) LFS-LTD was unsuccessfully induced in P60 mice that were knocked down 5-HT_{1A}Rs in serotonergic terminals (n = 8 slices/group; two-tailed Student's t-test; P=0.732).

Data were presented as mean ± s.e.m; *, $p < 0.05$.

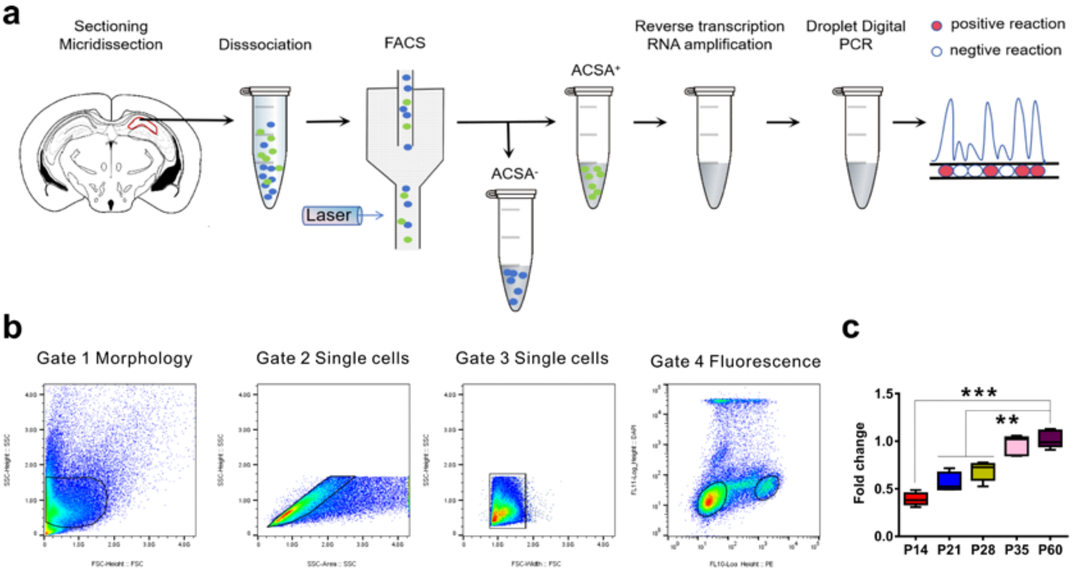

Supplementary Fig. 11

5-HT$_{1A}$R mRNA levels in hippocampal astrocytes from different aged mice.

(**a**) Detailed schematic diagram of the FACS-ddPCR process.

(**b**) Plots showing purification of astrocytes by FACS. ACSA^{+} or ACSA^{-} (ACSA-2: astrocyte cell surface antigen-2, a general astrocyte marker) cells were sorted by FACS.

(**c**) 5-HT$_{1A}$R mRNA levels in astrocytes from different age mice (n=5 mice/group; one-way ANOVA; $F_{(4, 20)}$=37.254, P=0.011).

Data show mean ± s.e.m..

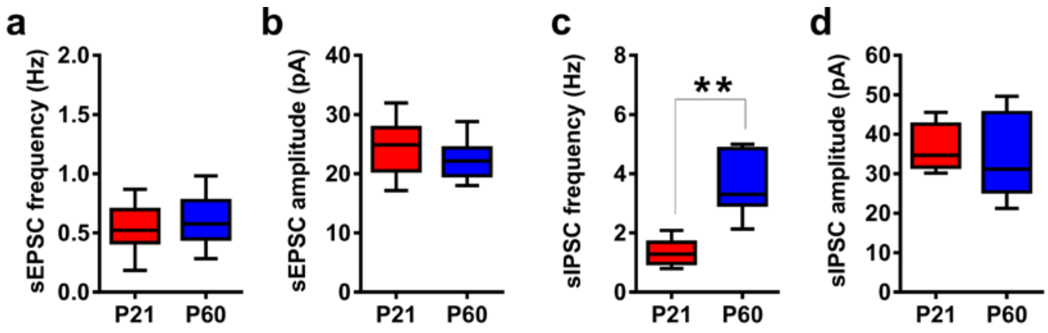

Supplementary Fig. 12

Increased GABAergic transmission in P60 mice.

(**a, b**) There was no difference of excitatory glutamatergic transmission between P21 and P60 mice (n = 10 cells from 5 slices from 4 mice, two-tailed Student's t-test, for a, P = 0.457, for b, P=0.682).

(**c, d**) Increased GABAergic transmission in P60 mice compared with that of P21 mcie (n = 10 cells from 5 slices from 4 mice, two-tailed Student's t-test, for c, P = 0.006, for d, P=0.724).

Data were presented as mean ± s.e.m.

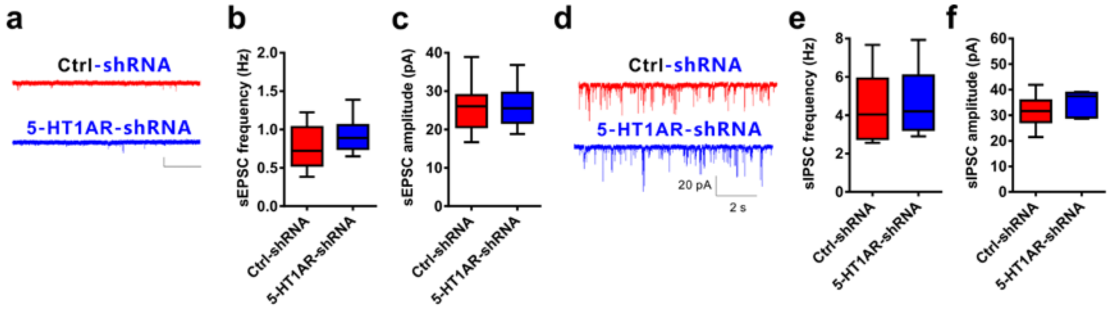

Supplementary Fig. 13

Knockdown of 5-HT$_{1A}$Rs in pyramidal neurons did not affect synaptic neurotransmission.

(**a-c**) Knockdown of 5-HT$_{1A}$R in pyramidal neurons did not affect excitatory glutamatergic transmission (n = 12 cells from 9 slices from 4 mice, two-tailed Student's t-test, for b, P = 0.484, for c, P=0.573). Scale bars: 20 pA, 2 s.

(**d-f**) Knockdown of 5-HT$_{1A}$R in pyramidal neurons did not affect inhibitory GABAergic transmission (n = 12 cells from 9 slices from 4 mice, two-tailed Student's t-test, for e, P = 0.264, for f, P=0.462). Scale bars: 20 pA, 2 s.

Data were presented as mean ± s.e.m.

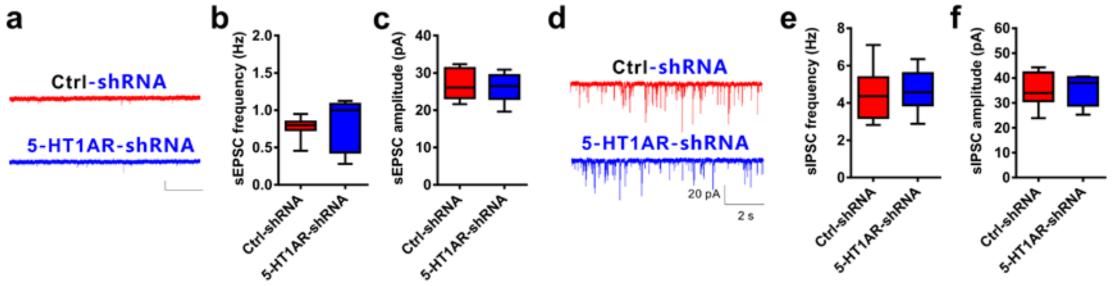

Supplementary Fig. 14

Knockdown of 5-HT$_{1A}$Rs in GABAergic neurons did not affect synaptic neurotransmission.

(**a-c**) Knockdown of 5-HT$_{1A}$R in GABAergic neurons did not affect excitatory glutamatergic transmission (n = 12 cells from 9 slices from 4 mice, two-tailed Student's t-test, for b, P = 0.765, for c, P=0.357). Scale bars: 20 pA, 2 s.

(**d-f**) Knockdown of 5-HT$_{1A}$R in GABAergic neurons did not affect inhibitory GABAergic transmission (n = 12 cells from 9 slices from 4 mice, two-tailed Student's t-test, for e, P = 0.523, for f, P=0.367). Scale bars: 20 pA, 2 s.

Data were presented as mean ± s.e.m.

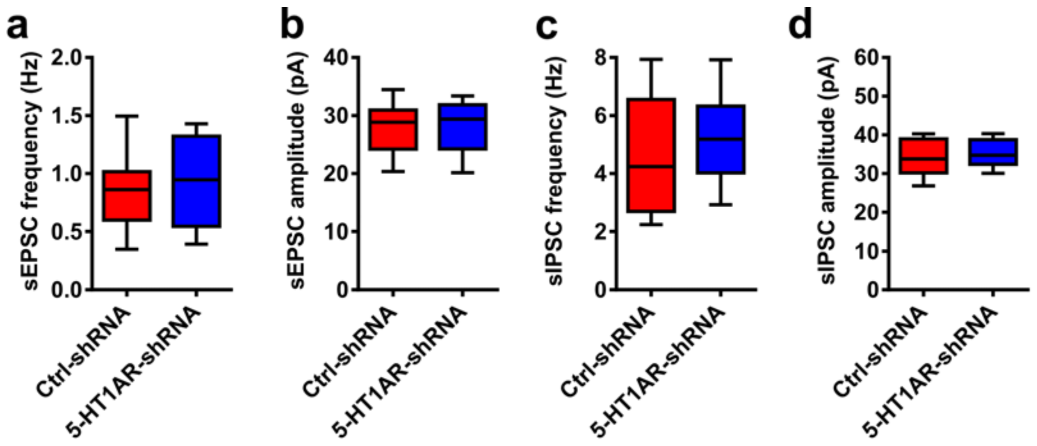

Supplementary Fig. 15

Knockdown of 5-HT$_{1A}$Rs in serotonergic terminals did not affect synaptic neurotransmission.

(**a, b**) Knockdown of 5-HT$_{1A}$R in serotonergic terminals did not affect excitatory glutamatergic transmission (n = 12 cells from 9 slices from 4 mice, two-tailed Student's t-test, for a, P = 0.284, for b, P=0.584).

(**c, d**) Knockdown of 5-HT$_{1A}$R in serotonergic terminals did not affect inhibitory GABAergic transmission (n = 12 cells from 9 slices from 4 mice, two-tailed Student's t-test, for c, P = 0.492, for d, P=0.543).

Data were presented as mean ± s.e.m.

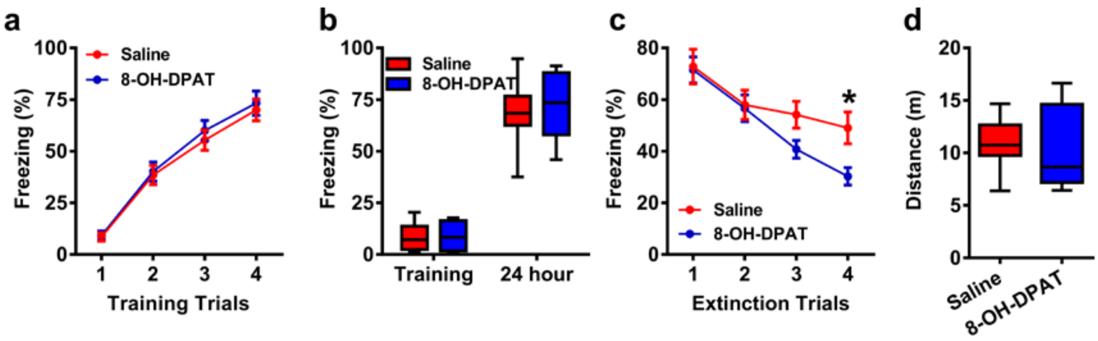

Supplementary Fig. 16

5-HT$_{1A}$R agonist facilitated fear memory extinction in P21 mice.

(**a-c**) Measurement of fear memory process in P21 mice after 8-OH-DPAT treatment (n=11 mice/group; for a, repeated measures two-way ANOVA, $F_{(1, 80)}$ = 14.536, P = 0.427; for b, two-tailed Student's t-test, P=0.332; for c, repeated measures two-way ANOVA, $F_{(1, 80)}$ = 35.634, P = 0.028).

(**d**) Locomoter activity was not affected after 8-OH-DPAT treatment (n =11 mice/group, two-tailed Student's t-test, P=0.634).

Data were presented as mean ± s.e.m. *, p < 0.05.

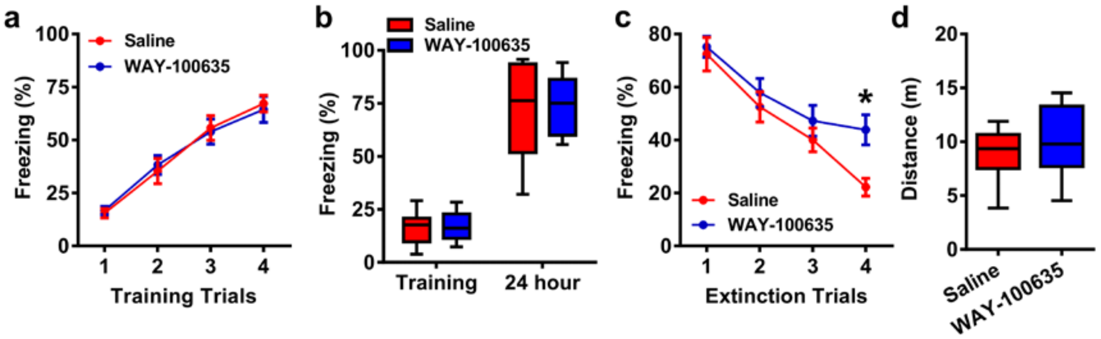

Supplementary Fig. 17

5-HT$_{1A}$R antagonist impaired fear memory extinction in P60 mice.

(**a-c**) Measurement of fear memory process in P60 mice after WAY-100635 treatment (n=12 mice/group; for a, repeated measures two-way ANOVA, $F_{(1, 88)}$ = 17.367, P = 0.683; for b, two-tailed Student's t-test, P=0.632; for c, repeated measures two-way ANOVA, $F_{(1, 88)}$ = 33.734, P = 0.034).

(**d**) Locomoter activity was not affected after WAY-100635 treatment (n =12 mice/group, two-tailed Student's t-test, P=0.463).

Data were presented as mean ± s.e.m. *, p < 0.05.

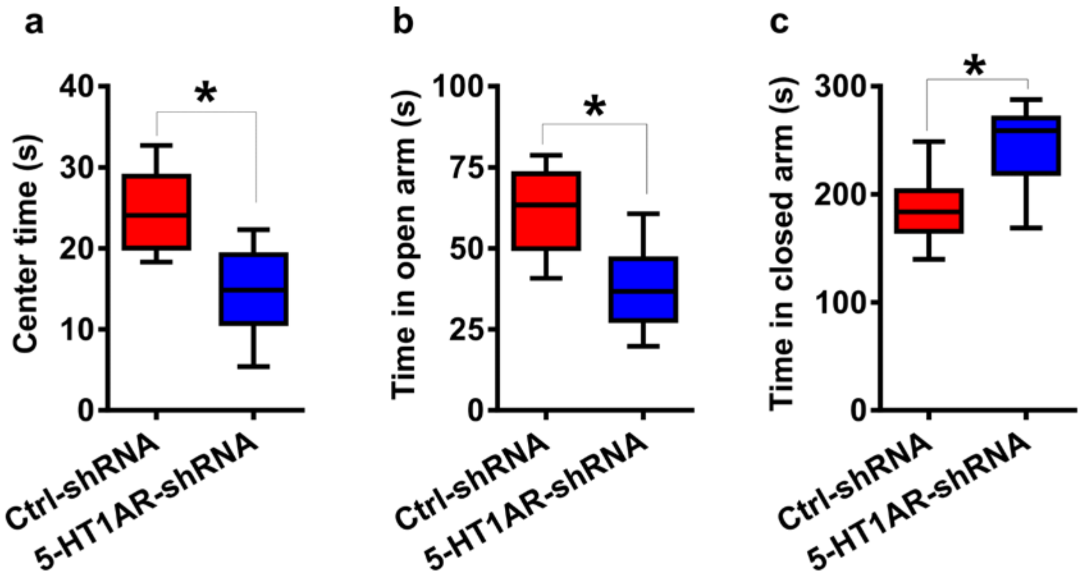

Supplementary Fig. 18

Knockdown of astrocytic 5-HT$_{1A}$Rs induced anxiety like behaviors.

(**a**) Mice with 5-HT$_{1A}$R -knockdown in astrocytes in CA1 spent less time in the central arena in the open field test (n=10-11 mice/group; two-tailed Student's t-test, P=0.024).

(**b, c**) In the EPM test, aldh1l1-CreER^{T2} mice injected with 5-HT$_{1A}$R shRNA spent less time in the open arm (b) and more time in the closed arms (c) (n =10-11 mice/group, two-tailed Student's t-test, for b: P=0.018; for c: P=0.012).

Data were presented as mean ± s.e.m. *, p < 0.05.

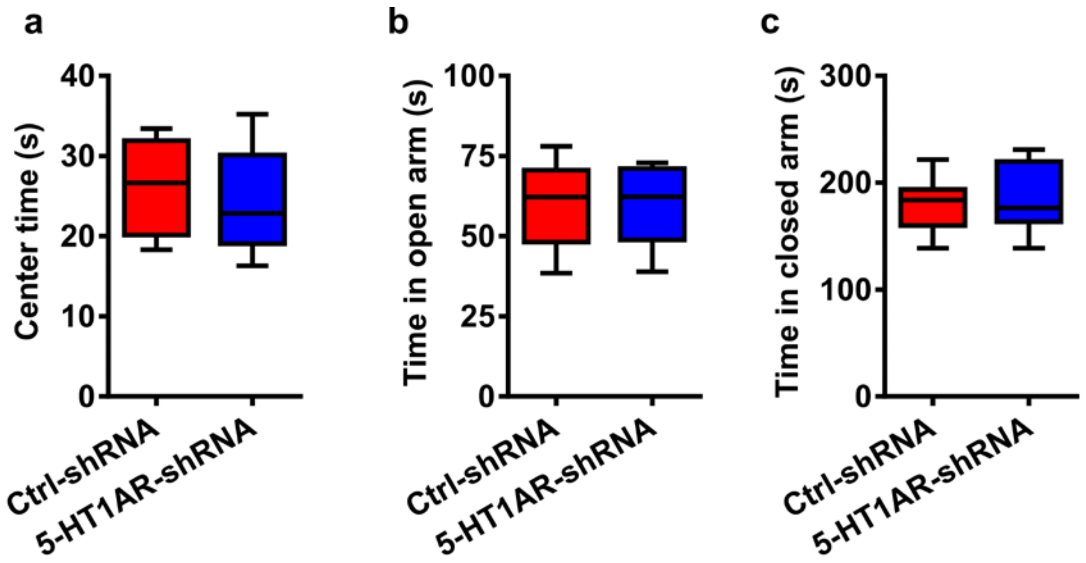

Supplementary Fig. 19

Knockdown of 5-HT_{1A}Rs in pyramidal neurons did not affect anxiety-like behaviors.

(**a**) Mice with 5-HT_{1A}R -knockdown in pyramidal neurons in CA1 spent the same time in the central arena in the open field test (n=11 mice/group; two-tailed Student's t-test, P=0.374).

(**b, c**) In the EPM test, CamKII-Cre mice injected with 5-HT_{1A}R shRNA spent the same time in the open (b) and closed arms (c) (n =11 mice/group, two-tailed Student's t-test, for b: P=0.138; for c: P=0.356).

Data were presented as mean ± s.e.m.

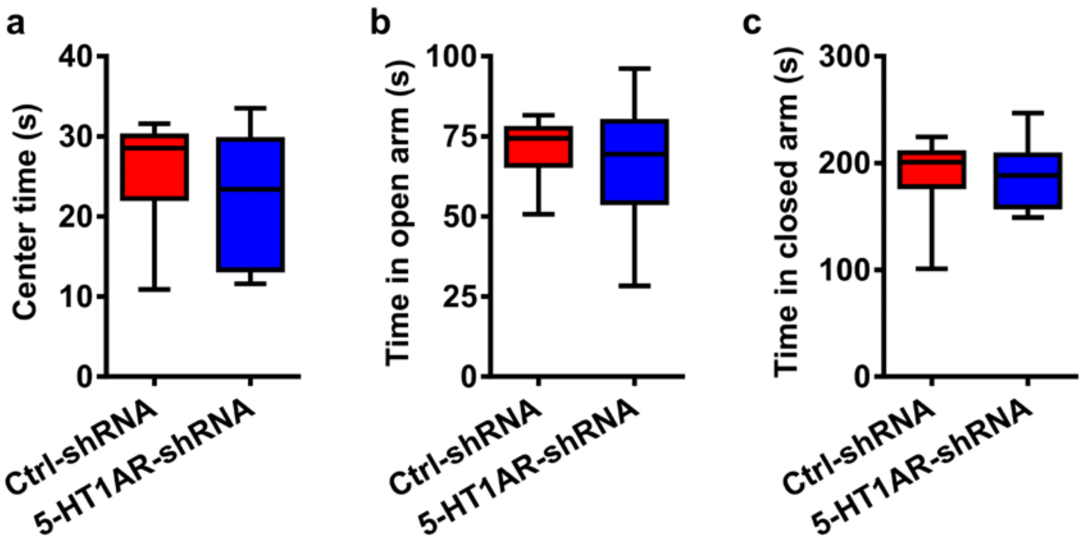

Supplementary Fig. 20

Knockdown of 5-HT$_{1A}$Rs in GABAergic neurons did not affect anxiety-like behaviors.

(**a**) Mice with 5-HT$_{1A}$R -knockdown in GABAergic neurons in CA1 spent the same time in the central arena in the open field test (n=10 mice/group; two-tailed Student's t-test, P=0.284).

(**b, c**) In the EPM test, GAD-Cre mice injected with 5-HT$_{1A}$R shRNA spent the same time in the open (b) and closed arms (c) (n =10 mice/group, two-tailed Student's t-test, for b: P=0.314; for c: P=0.267).

Data were presented as mean ± s.e.m.

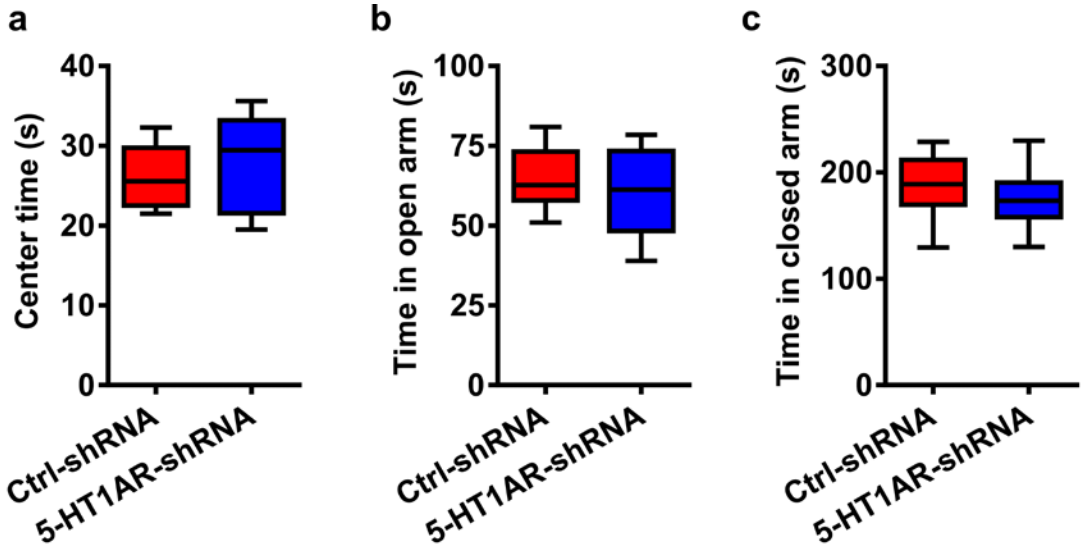

Supplementary Fig. 21

Knockdown of 5-HT$_{1A}$Rs in serotonergic terminals did not affect anxiety-like behaviors.

(**a**) Mice with 5-HT$_{1A}$R -knockdown in serotonergic terminals in CA1 spent the same time in the central arena in the open field test (n=10 mice/group; two-tailed Student's t-test, P=0.475).

(**b, c**) In the EPM test, sert-Cre mice injected with 5-HT$_{1A}$R shRNA spent the same time in the open (b) and closed arms (c) (n =10 mice/group, two-tailed Student's t-test, for b: P=0.344; for c: P=0.224).

Data were presented as mean ± s.e.m.
